# Blood biomarkers to improve dementia diagnostic accuracy: a cross-sectional analysis

**DOI:** 10.1186/s12877-026-07431-9

**Published:** 2026-05-01

**Authors:** Joseph Kwon, Megan Kirk Chang, Adam Gordon-Boyle, Sam Creavin, Lynne Hughes, Vanessa Raymont, Kamaldeep Bhui, Apostolos Tsiachristas

**Affiliations:** 1 Nuffield Department of Primary Care Health Sciences, University of Oxford, Radcliffe Primary Care Building, Radcliffe Observatory Quarter, Woodstock Road, Oxford, OX2 6GG England; 2 Medical Sciences Division, University of Oxford, John Radcliffe Hospital, Headley Way, Headington, Oxford, OX3 9DU England; 3 Centre for Academic Primary Care, Bristol Medical School, University of Bristol, 39 Whatley Road, Clifton,, Bristol, BS8 2PS England; 4 Global Alzheimer’s Platform Foundation, 4315 50th St. NW Ste 100 Unit 2623 , Washington, DC 20016 England; 5 Department of Psychiatry, University of Oxford, Warneford Hospital, Oxford, OX3 7JX England

**Keywords:** Mild cognitive impairment, Dementia, Diagnosis, Diagnostic accuracy, Blood biomarkers of Alzheimer’s disease

## Abstract

**Background:**

The recommended dementia diagnostic pathway comprises non-specialist assessment followed by specialist diagnosis. Given increasing resource constraints and existing inequalities in accessing specialist care, more accurate assessment in non-specialist settings may improve dementia management. This study assessed the diagnostic accuracy of blood biomarkers of Alzheimer’s disease (AD) and neurodegeneration for detecting probable AD (PAD) and mild cognitive impairment (MCI) with amyloid positivity (AP), particularly when they supplement current non-specialist practice of administering Mini-Mental State Examination (MMSE).

**Methods:**

We accessed data from the Bio-Hermes study which grouped participants as cognitively normal (n=417), MCI (n=312), and PAD (n=272). Blood biomarkers of AD and neurodegeneration included: amyloid-beta 42/40; phosphorylated-tau 181 (p-tau181); p-tau217; glial fibrillary acidic protein (GFAP); and neurofilament light (NfL). Biomarkers were added individually or as panel to MMSE to predict the following diagnostic outcomes: PAD; MCI or PAD (MCI-PAD); PAD with AP measured by positron emission tomography/cerebrospinal fluid (PAD-AP); and MCI-PAD with AP (MCI-PAD-AP). Accuracy was assessed using receiver operating characteristic (ROC) curve and area under ROC curve (AUC) following logistic regression, adjusted for covariates observable in general clinical setting (e.g., alcohol, smoking, functional impairment) and apolipoprotein E ε4 carrier status. Statistically significant differences in AUC were estimated by DeLong test. Subgroup analyses were conducted by age and race/ethnicity.

**Results:**

MMSE plus individual biomarkers or panels significantly improved accuracy to detect PAD-AP and MCI-PAD-AP versus MMSE alone: e.g., AUC for MMSE+p-tau217, adjusted for covariates, to detect MCI-PAD-AP was 0.928 versus 0.844 for MMSE alone (DeLong test for significance P<0.001); MMSE plus optimal panel comprising all five biomarkers achieved AUC of 0.939 (DeLong P<0.001 versus MMSE alone). AUC improvements from biomarker addition were smaller, sometimes not statistically significant, for PAD and MCI-PAD. Composition of optimal panel varied across subgroups: e.g., p-tau217 was included in the optimal panel for non-Hispanic White, while p-tau181 was included in the panel instead for non-White race/ethnicity.

**Conclusions:**

Blood biomarker supplementation of cognitive testing can improve detection of amyloid-positive MCI and dementia. This potentially supports an efficient and equitable dementia diagnostic pathway which contributes to the sustainable delivery of prospective amyloid-targeting therapies with proven safety, effectiveness and cost-effectiveness.

**Supplementary Information:**

The online version contains supplementary material available at 10.1186/s12877-026-07431-9.

## Background

Dementia is a clinical syndrome characterised by a range of cognitive and behavioural symptoms including memory loss and impaired ability to carry out activities of daily living [1]. It is the outcome of neurodegenerative diseases including Alzheimer’s disease (AD) which accounts for 60–80% of dementia cases [2]. Improving diagnosis of dementia is a public health priority to enable accurate identification of underlying neurodegenerative disease and related prognosis, lifestyle changes to modify risk factors exacerbating disease progression, and timely access to pharmacological treatments and care [3].

Since 2011, diagnosis of AD is to be informed by abnormal levels of biomarkers amyloid (Aβ) and tau – detected in vivo by lumbar puncture for cerebrospinal fluid (CSF), positron emission tomography (PET), and, most recently, plasma assay – rather than by clinical symptoms only [4–6]. These biomarkers help determine whether a given case of dementia or mild cognitive impairment (MCI) can be attributed to AD pathology, facilitating differential diagnosis and targeted treatment and support plans [7]. Specifically, amyloid positivity (AP) is a necessary condition for receiving amyloid-targeting treatments (ATTs), such as lecanemab and donanemab, both of which have received regulatory approvals in the US, UK and beyond [8, 9]. The latest AD drug development pipeline in 2024 shows 21 further amyloid-targeting agents being assessed in phase I-III trials, comprising 18% of all new disease-modifying agents being trialled [10].

To sustain the delivery of ATTs and other AD-targeted treatments, MCI and early AD patients with AP should be diagnosed efficiently and equitably. Nevertheless, the current dementia diagnostic pathways in developed countries are marked by significant inequalities in performance, particularly between ethnicity groups [11, 12]. These could be further exacerbated by the introduction of a complex diagnostic pathway to support the delivery of ATT [13].

The guideline-recommended dementia diagnostic pathway comprises assessment in non-specialist settings followed by differential diagnosis within a specialist setting [1]. Innovations to enhance the accuracy and speed of non-specialist assessment, such as at primary care consultations, are key to make the whole pathway efficient and equitable. Here, plasma assays of AD pathology and neurodegeneration – including phosphorylated tau 217 (p-tau217), p-tau181, Aβ42/Aβ40 ratio (Aβ42/40), glial fibrillary acidic protein (GFAP), and neurofilament light (NfL) – routinely implemented in non-specialist settings, offer such potential. Previous studies have demonstrated the capacity of above biomarkers to predict PET- or CSF-measured AP with high accuracy [6, 7, 14–16], with p-tau217 generally outperforming other biomarkers and combination of biomarkers outperforming single biomarkers [17]. The use of blood biomarkers would likely supplement current practice in non-specialist settings, including the administration of validated cognitive screening tools such as the Mini-Mental State Examination (MMSE) [18], consideration of clinical history, and routine laboratory investigations to rule out reversible causes of cognitive decline [1].

To our knowledge, no previous study has explored the performance of blood biomarkers supplemented to current practice in diagnosing MCI or mild dementia with and without AP relative to current practice alone. This study aims to assess whether the addition of blood biomarkers to current practice, represented by application of MMSE, generates significant improvements in diagnostic accuracy relative to current practice alone.

## Methods

Reporting for this study followed the Standards for Reporting of Diagnostic Accuracy Studies (STARD) [19]. See the Appendix for the completed STARD checklist.

### Data

We used data from the Bio-Hermes study [14] which enrolled 1,001 individuals aged 60 to 85 years from community-based populations in the US across 17 research sites between April 2021 and November 2022. Participants were grouped by clinical judgement – cognitively normal (*n* = 417), MCI (*n* = 312), and probable AD (PAD) (*n* = 272) – of whom 956 (95.5%) completed PET (*n* = 945) or CSF (*n* = 11) measurement of Aβ level. Blood samples were collected at the first study visit from which the following biomarkers were obtained: Aβ40, Aβ42, Aβ42/40, t-tau, p-tau181, p-tau217, GFAP, NfL, and genetic biomarker apolipoprotein E ε4 (APOE4) carrier status. The blood biomarkers were processed at different laboratories and were sourced in our study as follows: Aβ40 and Aβ42 from C_2_N; p-tau181, GFAP, NfL and APOE4 from Roche; and p-tau217 from Eli Lilly.

The data also included age (continuous); sex (male vs. female); race and ethnicity, combined into a binary indicator (non-Hispanic White vs. other); blood pressure (high vs. low, see Table A.1 in the Appendix for thresholds); weight (overweight/obese vs. normal/underweight based on body mass index, see Table A.1); alcohol consumption (current/past consumption vs. none); tobacco consumption (current/past consumption vs. none); depression based on Geriatric Depression Scale and clinical history (yes vs. no, see Table A.1); APOE4 status (carrier vs. non-carrier); and Functional Activities Questionnaire (FAQ; continuous with range 0–30, with higher score implying greater functional impairment) [20]. These variables were included as covariates in the logistic regression models used to predict diagnostic outcomes. It was assumed that APOE4 status is unknown in strategies not employing blood biomarkers. Years of education and blood cholesterol were not used as covariates due to high rates of missing data (> 10% of sample).

### Diagnostic outcomes

The following four diagnostic outcomes were defined based on clinical judgement and PET/CSF-measured AP:

1. PAD.
2. MCI or PAD (MCI-PAD).
3. PAD with AP (PAD-AP).
4. MCI-PAD with AP (MCI-PAD-AP).

Outcomes (1) and (2) will also be referred as ‘all-cause’ PAD and MCI-PAD since they incorporate PAD and MCI with and without AP. We used the binary indicator for AP as the Bio-Hermes study team contained in the shared dataset [14]. Clinical judgement of PAD and MCI was made by investigators of the Bio-Hermes study when participants were screened for study eligibility [14]. Participants were diagnosed with PAD according to the National Institute on Aging and Alzheimer’s Association (NIA-AA) criteria [5] and verified through medical records or according to the following screening results: MMSE 20–24 (investigator’s clinical judgement when MMSE < 20); Rey Auditory Verbal Learning Test (RAVLT) delayed recall score ≥ 1 standard deviation (SD) below age- and race-adjusted mean; and evidence of functional decline or impairment according to investigator’s judgement, participant partner report, or FAQ score. MCI was similarly diagnosed according to the NIA-AA criteria and verified through the screening results where required: MMSE 24–30; RAVLT delayed recall score ≥ 1 SD below age- and race-adjusted mean; and evidence of minimal/mild functional impairment but preservation of functional independence. It was not reported what proportions of the PAD and MCI diagnoses were verified through the screening results [14].

### Diagnostic tests and covariates

MMSE – unadjusted or adjusted for covariates as described below – was assumed to be the reference case test in non-specialist setting. This was compared to:

1. MMSE plus individual blood biomarkers: Aβ42/40 ratio; p-tau181; p-tau217; GFAP; and NfL.
2. MMSE plus multiple blood biomarkers (i.e., biomarker panels).

To identify the optimal biomarker panel, all five biomarkers were initially included in the logistic regression, then those that were not significantly associated with the outcome at 95% significance level were sequentially excluded from the final panel, with the logistic model fit (measured as described below) being checked at every change. As supplementary analyses, scenarios of individual biomarkers being used without MMSE were evaluated.

Previous analysis found no statistically significant difference in t-tau level between amyloid positive and negative subgroups, and hence t-tau was not used as a biomarker [14]. The methods for processing the variables, including imputations of biomarker values and removal of outliers, are outlined in Table A.1 in the Appendix. Aβ42/40 (range 0.07–0.15) and p-tau217 (range 0.001–1.39) values were multiplied by 100 to ease interpretation of their coefficients in logistic regressions.

### Statistical analyses

All analyses were run on R version 4.1.2 available on AD Workbench hosted by AD Data Initiative [21]. Complete cases, except for imputations of p-tau181 and p-tau217 (see Table A.1 in the Appendix), were used for all analyses. Analysis of variance (ANOVA) was used to identify statistically significant univariate difference in mean values of continuous variables across outcome categories and chi-square test for categorical variables. Logistic regressions were performed with individual or multiple tests as explanatory variables and adjusted for covariates where relevant.

The receiver operating characteristic (ROC) curve and area under the ROC curve (AUC) value were derived from each logistic regression using the pROC function in R. DeLong test [22] was used to identify statistically significant difference in AUC values between diagnostic strategies. When using the DeLong test to compare two nested models (i.e., between models adjusted and unadjusted for covariates), only covariates that are significantly associated with the outcome were included to preclude the loss of statistical power [23]. To identify the significantly associated covariates, all covariates were first included in the logistic regression; those that were not independently associated with the outcome at 95% significance level were excluded; the regression was then re-run with remaining covariates. The model fit was assessed by Akaike information criterion (AIC) and Bayesian information criterion (BIC) values, divided by sample size, with lower values indicating better fit. For each diagnostic strategy, the optimal cut-off threshold was estimated by Youden’s index [24]; the sensitivity and specificity values associated with the optimal threshold were estimated. It should be noted that because MMSE and FAQ measures were used to verify clinical judgement of PAD and MCI according to the NIA-AA criteria, models that included the same measures as predictors may overestimate their accuracy – this is discussed as a study limitation below.

### Subgroup analyses

Subgroups were defined by age (below vs. above sample median) and race/ethnicity (non-Hispanic White vs. other). For each diagnostic outcome and subgroup, the optimal biomarker panel was identified as above. MMSE plus the optimal panel was then compared to MMSE alone, both models adjusted for covariates. The model fit, AUC, sensitivity and specificity were estimated. DeLong test was not applied to compare the AUC values from different subgroups since the models are not nested.

## Results

### Sample characteristics

Table 1 shows the sample characteristics by outcome categories. Notably, 38.7% (96 of 248) of those with clinical diagnosis of PAD were amyloid negative, indicating that clinical judgement has poor specificity in diagnosing AD subtype of dementia. Participants with PAD or MCI-PAD, all-cause or with AP, were significantly more likely to be APOE4 carriers and have lower MMSE and higher FAQ scores than those without. There were significant differences in all blood biomarkers between outcome subgroups. There were significantly lower proportions of non-Hispanic White individuals among those with all-cause PAD or MCI-PAD than those without, while the proportions were similar between PAD-AP and MCI-PAD-AP subgroups. Table A.2 in the Appendix shows the sample characteristics by clinical categories of cognitively normal, MCI and PAD.

**Table 1:** Sample characteristics by diagnosis and amyloid positivity status

	PAD			MCI-PAD			PAD-AP			MCI-PAD-AP			Total sample [*n* = 928]
	No [*n* = 680]	Yes [*n* = 248]	*P*-value	No [*n* = 394]	Yes [*n* = 534]	*P*-value	No [*n* = 776]	Yes [*n* = 152]	*P*-value	No [*n* = 673]	Yes [*n* = 255]	*P*-value	
Age (years), mean (SD)	71.1 (6.7)	74.4 (6.1)	**<** 0.001	70.3 (6.4)	73.2 (6.6)	**<** 0.001	71.4 (6.7)	75.0 (5.7)	**< **0.001	71.0 (6.6)	74.6 (6.0)	**< 0.001**	72.0 (6.7)
Female, n (%)	397 (58.4)	131 (52.8)	0.150	241 (61.2)	287 (53.7)	0.029	450 (58.0)	78 (51.3)	0.153	395 (58.7)	133 (52.2)	0.085	528 (56.9)
Non-Hispanic White, n (%)	541 (79.6)	169 (68.1)	**<** 0.001	320 (81.2)	390 (73.0)	0.005	598 (77.1)	112 (73.7)	0.427	514 (76.4)	196 (76.9)	0.944	710 (76.5)
High blood pressure,^a^ n (%)	223 (32.8)	107 (43.1)	0.005	132 (33.5)	198 (37.1)	0.291	268 (34.5)	62 (40.8)	0.168	240 (35.7)	90 (35.3)	0.978	330 (35.6)
Overweight/ obese,^b^ n (%)	460 (67.6)	145 (58.5)	0.012	278 (70.6)	327 (61.2)	0.004	524 (67.5)	81 (53.3)	0.001	464 (68.9)	141 (55.3)	**< **0.001	605 (65.2)
Alcohol use, n (%)	501 (73.7)	157 (63.3)	0.003	293 (74.4)	365 (68.4)	0.055	556 (71.6)	102 (67.1)	0.303	475 (70.6)	183 (71.8)	0.784	658 (70.9)
Tobacco use, n (%)	265 (39.0)	91 (36.7)	0.579	143 (36.3)	213 (39.9)	0.296	297 (38.3)	59 (38.8)	0.972	260 (38.6)	96 (37.6)	0.841	356 (38.4)
Depression,^c^ n (%)	192 (28.2)	90 (36.3)	0.023	102 (25.9)	180 (33.7)	0.013	226 (29.1)	56 (36.8)	0.073	193 (28.7)	89 (34.9)	0.078	282 (30.4)
APOE4 carrier, n (%)	236 (34.7)	113 (45.6)	0.003	128 (32.5)	221 (41.4)	0.007	250 (32.2)	99 (65.1)	**< **0.001	184 (27.3)	165 (64.7)	**<** 0.001	349 (37.6)
Aβ42/40,^d^ mean (SD)	9.756 (0.978)	9.578 (0.985)	0.015	9.844 (0.968)	9.608 (0.982)	**< **0.001	9.812 (0.982)	9.178 (0.796)	**<** 0.001	9.946 (0.962)	9.083 (0.729)	**< **0.001	9.709 (0.983)
p-tau181 (pg/mL), mean (SD)	0.967 (0.468)	1.387 (0.656)	**< **0.001	0.900 (0.380)	1.212 (0.625)	**< **0.001	0.970 (0.473)	1.638 (0.616)	**< **0.001	0.900 (0.415)	1.553 (0.604)	**< **0.001	1.079 (0.557)
p-tau217 (U/mL),^d^ mean (SD)	19.6 (15.2)	38.6 (30.5)	**< **0.001	17.0 (9.8)	30.2 (26.5)	**< **0.001	19.8 (16.6)	49.3 (29.0)	**<** 0.001	17.2 (13.4)	44.4 (27.6)	**< **0.001	24.6 (22.1)
GFAP (pg/mL), mean (SD)	102.4 (54.0)	137.4 (65.8)	**<** 0.001	94.9 (47.1)	124.1 (64.3)	**<** 0.001	102.2 (53.4)	160.3 (64.6)	**< **0.001	95.3 (46.9)	155.1 (66.6)	**<** 0.001	111.7 (59.4)
NfL (pg/mL), mean (SD)	3.138 (1.853)	4.256 (2.515)	**<** 0.001	2.839 (1.540)	3.877 (2.350)	**< **0.001	3.185 (1.914)	4.719 (2.552)	**< **0.001	3.091 (1.906)	4.349 (2.337)	**< **0.001	3.436 (2.108)
MMSE total, mean (SD)	27.9 (1.8)	23.3 (2.5)	**< **0.001	28.4 (1.5)	25.4 (3.0)	**< **0.001	27.4 (2.3)	22.8 (2.5)	**<** 0.001	27.5 (2.3)	24.5 (3.0)	**< **0.001	26.7 (2.9)
FAQ, mean (SD)	1.9 (3.4)	9.1 (6.4)	**< **0.001	0.7 (1.5)	6.2 (6.1)	**< **0.001	2.7 (4.5)	9.7 (6.1)	**< **0.001	2.4 (4.3)	7.7 (6.2)	**< **0.001	3.9 (5.4)

*Abbreviation*:*AP* Amyloid positivity, *APOE4,* Apolipoprotein E allele 4, *DBP* Diastolic blood pressure, *FAQ* Functional Activities Questionnaire, *GFAP* Glial fibrillary acidic protein, *MCI* Mild cognitive impairment, *MMSE* Mini-Mental State Examination, *NfL* Neurofilament light; *PAD* Probable Alzheimer’s disease, *SBP* Systolic blood pressure, *SD* Standard deviation

^a^High if DBP ≥ 90 mmHg or SBP ≥ 140 mmHg

^b^Obese if BMI ≥ 30; overweight if 25 ≤ BMI < 30

^c^Depressed if Geriatric Depression Scale (GDS) ≥ 5 or has ongoing (major) depression according to medical history

^d^Multiplied by 100 for ease of interpreting logistic regression coefficients

### Diagnostic accuracy of MMSE plus individual biomarkers

Table 2 shows by outcome the AUC values for MMSE plus individual blood biomarkers, unadjusted and adjusted for significantly associated covariates. Tables A.3–6 in the Appendix show the logistic regression coefficients and other test accuracy metrics. For outcomes PAD and MCI-PAD, additions of individual biomarkers generally brought statistically significant improvement in AUC value relative to unadjusted MMSE, but not to adjusted MMSE. By contrast, additions of individual biomarkers brought significant improvements in AUC value to both unadjusted and adjusted MMSE for PAD-AP and MCI-PAD-AP outcomes: e.g., AUC of adjusted MMSE was 0.844 for MCI-PAD-AP, which increased to 0.928 when p-tau217 was added. Adjustment for covariates, particularly FAQ, consistently improved AUC values for all outcomes. PAD-AP was consistently associated with age, APOE4 status, tobacco use, and FAQ when individual biomarkers were included (Table A.5), and MCI-PAD-AP with age, alcohol use, APOE4 status, and FAQ (Table A.6). Figure A.1 in the Appendix shows the ROC curves of all test strategies involving individual biomarkers by outcome.

**Table 2:** AUC values for MMSE plus individual blood biomarkers

	MMSE	MMSE + Aβ42/40	MMSE + *p*-tau181	MMSE + *p*-tau217	MMSE + GFAP	MMSE + NfL
Outcome: PAD. AUC (DeLong test *P*-value)^a^						
(1) Unadjusted	0.920 (reference)	0.919 (*P* = 0.541)	**0.925** **(** ***P*** **= 0.023)**	**0.927** **(** ***P*** **= 0.021)**	**0.924** **(** ***P*** **= 0.041)**	**0.925** **(** ***P*** **= 0.007)**
(2) Adjusted^b^	0.950 (reference)	0.952 (*P* = 0.146)	0.951 (*P* = 0.297)	0.952 (*P* = 0.144)	0.950 (*P* = 0.221)	0.952 (*P* = 0.152)
DeLong test *P*-value for (1) vs. (2)	***P*** ** < 0.001**	***P*** ** < 0.001**	***P*** ** < 0.001**	***P*** ** < 0.001**	***P*** ** < 0.001**	***P*** ** < 0.001**
Outcome: MCI-PAD. AUC (DeLong test *P*-value)^a^						
(1) Unadjusted	0.807 (reference)	**0.813** **(** ***P*** **= 0.031)**	**0.819** **(** ***P*** **= 0.014)**	**0.819** **(** ***P*** **= 0.009)**	**0.815** **(** ***P*** **= 0.026)**	**0.820** **(** ***P*** **= 0.005)**
(2) Adjusted^b^	0.876 (reference)	0.878 (*P* = 0.106)	**0.881** **(** ***P*** **= 0.033)**	0.879 (*P* = 0.137)	0.879 (*P* = 0.080)	**0.881** **(** ***P*** **= 0.018)**
DeLong test *P*-value for (1) vs. (2)	***P*** ** < 0.001**	***P*** ** < 0.001**	***P*** ** < 0.001**	***P*** ** < 0.001**	***P*** ** < 0.001**	***P*** ** < 0.001**
Outcome: PAD-AP. AUC (DeLong test *P*-value)^a^						
(1) Unadjusted	0.903 (reference)	**0.922** **(** ***P*** **= 0.005)**	**0.933** **(** ***P*** **< 0.001)**	**0.934** **(** ***P*** **< 0.001)**	**0.923** **(** ***P*** **< 0.001)**	**0.915** **(** ***P*** **= 0.002)**
(2) Adjusted^b^	0.929 (reference)	**0.950** **(** ***P*** **< 0.001)**	**0.956** **(** ***P*** **< 0.001)**	**0.958** **(** ***P*** **< 0.001)**	**0.953** **(** ***P*** **< 0.001)**	**0.949** **(** ***P*** **< 0.001)**
DeLong test *P*-value for (1) vs. (2)	***P*** ** < 0.001**	***P*** ** < 0.001**	***P*** ** < 0.001**	***P*** ** < 0.001**	***P*** ** < 0.001**	***P*** ** < 0.001**
Outcome: MCI-PAD-AP. AUC (DeLong test *P*-value)^a^						
(1) Unadjusted	0.787 (reference)	**0.875** **(** ***P*** **< 0.001)**	**0.882** **(** ***P*** **< 0.001)**	**0.898** **(** ***P*** **< 0.001)**	**0.854** **(** ***P*** **< 0.001)**	**0.808** **(** ***P*** **< 0.001)**
(2) Adjusted^b^	0.844 (reference)	**0.915** **(** ***P*** **< 0.001)**	**0.922** **(** ***P*** **< 0.001)**	**0.928** **(** ***P*** **< 0.001)**	**0.910** **(** ***P*** **< 0.001)**	**0.889** **(** ***P*** **< 0.001)**
DeLong test *P*-value for (1) vs. (2)	***P*** ** < 0.001**	***P*** ** < 0.001**	***P*** ** < 0.001**	***P*** ** < 0.001**	***P*** ** < 0.001**	***P*** ** < 0.001**

*Abbreviation*: *AP* Amyloid positivity, *APOE4* Apolipoprotein E allele 4, *AUC* Area under the receiver operating characteristic curve, *FAQ* Functional Activities Questionnaire;, *GFAP* Glial fibrillary acidic protein, *MCI* Mild cognitive impairment, *MMSE* Mini-Mental State Examination, *NfL* Neurofilament light, *PAD* Probable Alzheimer’s disease

^a^Reference case for the DeLong test for AUC differences across the columns is the AUC for MMSE model (unadjusted or adjusted) for each outcome. Bold if *P* < 0.05

^b^Among covariates age, sex, ethnicity, overweight/obese, alcohol use, tobacco use, high blood pressure, depression, APOE4 carrier status and FAQ, each model is adjusted for those that are significantly associated with the outcome at 95% significance level. For MMSE models without blood biomarkers, APOE4 carrier status is excluded as covariate since it is assumed that this information is unavailable without blood draw

### Diagnostic accuracy of MMSE plus biomarker panels

**Fig. 1:**
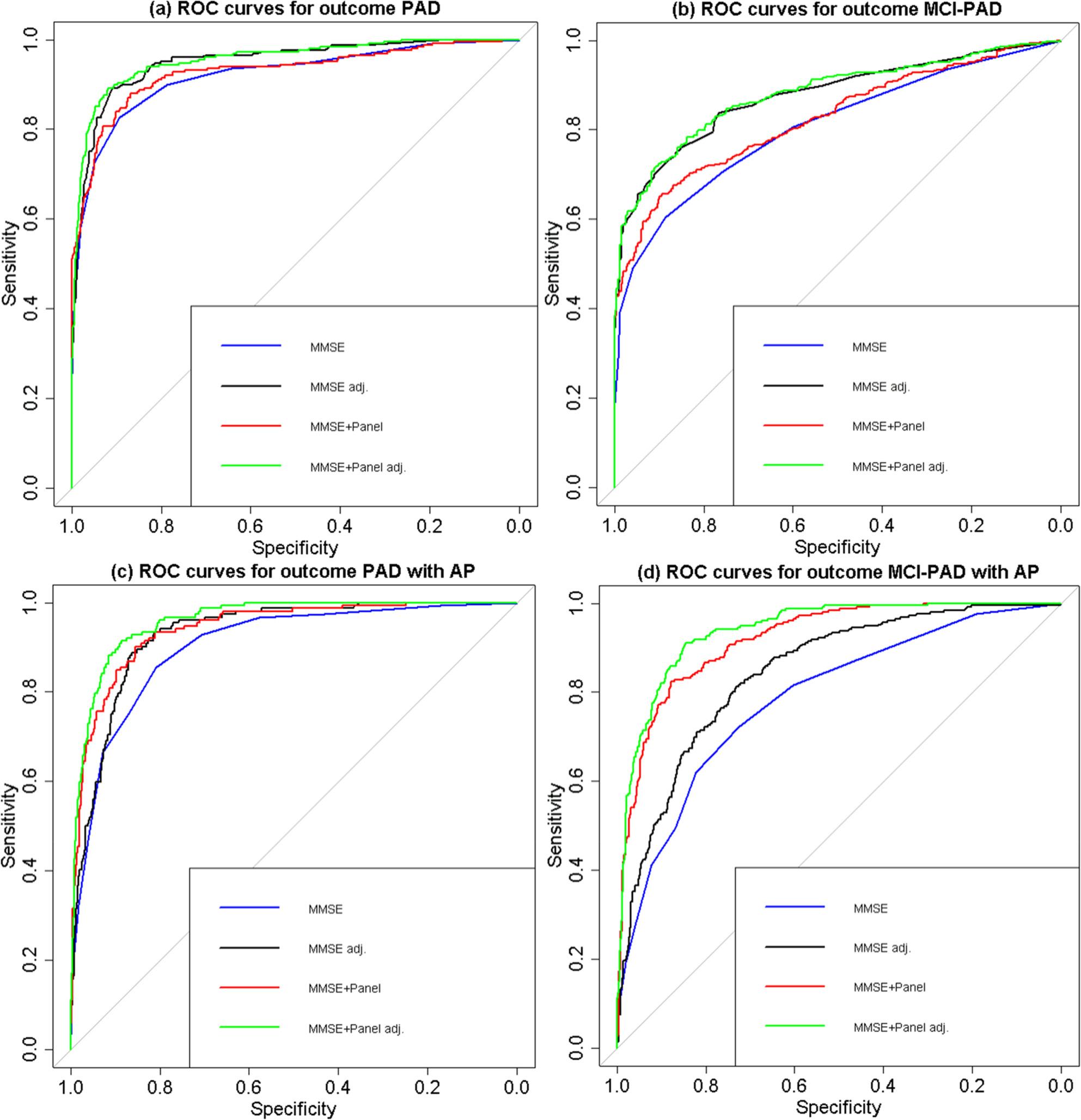
ROC curves for MMSE plus biomarker panels by outcome. Abbreviation: adj.: adjusted; AP: amyloid positive; GFAP: glial fibrillary acidic protein; MCI: mild cognitive impairment; MMSE: Mini-Mental State Examination; NfL: neurofilament light; PAD: probable Alzheimer’s disease; ROC: receiver operating characteristic

Table 3 shows by outcome the AUC values for MMSE combined with the optimal biomarker panel. Table A.7 in the Appendix shows the regression coefficients and accuracy metrics. All biomarker panels included p-tau217. Panels for PAD-AP and MCI-PAD-AP consistently included p-tau181. GFAP was included in both panels (unadjusted and adjusted) for MCI-PAD-AP, while NfL was included in the adjusted panel for MCI-PAD-AP. This meant that all five blood biomarkers were included in the adjusted panel for MCI-PAD-AP. The magnitude of the improvements in AUC from the addition of biomarker panels was greatest for MCI-PAD-AP, increasing from 0.787 to 0.919 when unadjusted for covariates and from 0.844 to 0.939 when adjusted. Figure 1 shows the corresponding ROC curves by outcome. FAQ was consistently included in the best-fit regression models for all outcomes, while age was included in all models except for MCI-PAD. APOE4 status was included in the models for PAD-AP and MCI-PAD-AP. Tobacco use was included in the model for PAD-AP, while alcohol use was included for MCI-PAD-AP.

**Table 3:** MMSE and biomarker panel accuracy by outcome

	MMSE	MMSE + Biomarker panel	MMSE	MMSE + Biomarker panel
	Outcome: PAD		Outcome: MCI-PAD	
(1) Unadjusted (DeLong test *P*-value vs. MMSE)	AUC = 0.920 (reference)	AUC = 0.927 (*P* = 0.026) Panel: Aβ42/40 p-tau217	AUC = 0.807 (reference)	AUC = 0.825 (*P* = 0.002) Panel: p-tau217 NfL
(2) Adjusted^a^ (DeLong test *P*-value vs. MMSE)	AUC = 0.950 (reference)	AUC = 0.954 (*P* = 0.036) Panel: Aβ42/40 p-tau217	AUC = 0.876 (reference)	AUC = 0.879 (*P* = 0.137) Panel: p-tau217
DeLong test *P*-value for (1) vs. (2)	*P* < 0.001	*P* < 0.001	*P* < 0.001	*P* < 0.001
	Outcome: PAD-AP		Outcome: MCI-PAD-AP	
(1) Unadjusted (DeLong test *P*-value vs. MMSE)	AUC = 0.903 (reference)	AUC = 0.941 (*P* < 0.001) Panel: Aβ42/40 p-tau181 p-tau217	AUC = 0.787 (reference)	AUC = 0.919 (*P* < 0.001) Panel: Aβ42/40 p-tau181 p-tau217 GFAP
(2) Adjusted^a^ (DeLong test *P*-value vs. MMSE)	AUC = 0.929 (reference)	AUC = 0.959 (*P* < 0.001) Panel: p-tau181 p-tau217	AUC = 0.844 (reference)	AUC = 0.939 (*P* < 0.001) Panel: Aβ42/40 p-tau181 p-tau217 GFAP NfL
DeLong test *P*-value for (1) vs. (2)	*P* < 0.001	*P* = 0.002	*P* < 0.001	*P* < 0.001

*Abbreviation*: *AP* Amyloid positivity, *APOE4* Apolipoprotein E allele 4, *AUC* Area under the receiver operating characteristic curve, *GFAP* Glial fibrillary acidic protein, *MCI* Mild cognitive impairment, *MMSE* Mini-Mental State Examination, *NfL* Neurofilament light, *PAD* Probable Alzheimer’s disease

^a^Among covariates age, sex, ethnicity, overweight/obese, alcohol use, tobacco use, high blood pressure, depression, and APOE4 carrier status, each model is adjusted for those that are significantly associated with the outcome at 95% significance level. For MMSE models without blood biomarkers, APOE4 carrier status is excluded as covariate since it is assumed that this information is unavailable without blood draw

### Subgroup analyses

Table 4 shows the subgroup analysis results; Tables A.8–11 in the Appendix show the corresponding regression coefficients and accuracy metrics. At subgroup levels, additions of biomarker panels for outcomes PAD and MCI-PAD no longer generated statistically significant improvement in AUC. For outcomes PAD-AP and MCI-PAD-AP, the significant improvements in AUC remained, but there were variations across subgroups on which biomarkers were included in the optimal panel. For instance, the optimal panels for outcome PAD-AP included p-tau217 for non-Hispanic White and p-tau181 for other race/ethnicity; those for outcome MCI-PAD-AP included Aβ42/40, p-tau217, GFAP and NfL for non-Hispanic White and Aβ42/40 and p-tau181 for other race/ethnicity. Thus, p-tau181 generally replaced p-tau217 within the optimal biomarker panel for the other race/ethnicity subgroup relative to non-Hispanic White. The covariates included in best-fit models also differed by subgroup (Tables A.8–11). Furthermore, the optimal cut-offs or threshold probability scores calculated from the Youden index differed visibly across subgroups (Tables A.8–11). Generally, the subgroup aged more than the median age and the other race/ethnicity subgroup had higher threshold probabilities than their younger and non-Hispanic White counterparts.

**Table 4:** MMSE and biomarker panel accuracy by subgroup and outcome

Outcome	Age ≤ 72		Age > 72		Non-Hispanic White		Other race/ethnicity	
	MMSE, adjusted	MMSE + panel, adjusted	MMSE, adjusted	MMSE + panel, adjusted	MMSE, adjusted	MMSE + panel, adjusted	MMSE, adjusted	MMSE + panel, adjusted
PAD (DeLong test *P*-value vs. MMSE, adjusted)	AUC = 0.952 (reference)	AUC = 0.958 (*P* = 0.064) Panel: Aβ42/40 p-tau217	AUC = 0.944 (reference)	AUC = 0.945 (*P* = 0.676) Panel: Aβ42/40	AUC = 0.952 (reference)	AUC = 0.954 (*P* = 0.243) Panel: Aβ42/40 p-tau217	AUC = 0.954 (reference)	AUC = 0.961 (*P* = 0.057) Panel: Aβ42/40 p-tau181
MCI-PAD (DeLong test *P*-value vs. MMSE, adjusted)	AUC = 0.861 (reference)	AUC = 0.861 (*P* = 1.000) Panel: No biomarker	AUC = 0.887 (reference)	AUC = 0.892 (*P* = 0.200) Panel: p-tau217	AUC = 0.871 (reference)	AUC = 0.872 (*P* = 0.775) Panel: p-tau217	AUC = 0.904 (reference)	AUC = 0.909 (*P* = 0.518) Panel: NfL
PAD-AP (DeLong test *P*-value vs. MMSE, adjusted)	AUC = 0.943 (reference)	AUC = 0.974 (*P* = 0.002) Panel: p-tau217	AUC = 0.905 (reference)	AUC = 0.937 (*P* = 0.001) Panel: p-tau217	AUC = 0.937 (reference)	AUC = 0.961 (*P* = 0.001) Panel: p-tau217	AUC = 0.914 (reference)	AUC = 0.956 (*P* = 0.005) Panel: p-tau181
MCI-PAD-AP (DeLong test *P*-value vs. MMSE, adjusted)	AUC = 0.865 (reference)	AUC = 0.953 (*P* < 0.001) Panel: Aβ42/40 p-tau217	AUC = 0.805 (reference)	AUC = 0.923 (*P* < 0.001) Panel: Aβ42/40 p-tau217 GFAP NfL	AUC = 0.840 (reference)	AUC = 0.943 (*P* < 0.001) Panel: Aβ42/40 p-tau217 GFAP NfL	AUC = 0.824 (reference)	AUC = 0.932 (*P* < 0.001) Panel: Aβ42/40 p-tau181

*Abbreviation*: *AP* Amyloid positivity, *AUC* Area under the receiver operating characteristic curve, *GFAP* Glial fibrillary acidic protein, *MCI* Mild cognitive impairment, *MMSE* Mini-Mental State Examination, *NfL* Neurofilament light, *PAD* Probable Alzheimer’s disease

## Discussion

This study evaluated the predictive accuracy of blood biomarkers of AD and neurodegeneration in detecting key dementia diagnostic outcomes. Individual biomarkers or biomarker panels added to MMSE significantly outperformed MMSE alone in detecting amyloid positive individuals with MCI or mild dementia who comprise the main target population for ATTs. Blood biomarkers could therefore be used alongside current cognition testing in non-specialist settings to triage for this population. Which biomarker comprised the optimal panel varied by subgroup, suggesting the need for tailored strategy.

This evidence makes a timely contribution to discussions on the sustainable delivery of prospective ATTs with proven safety, effectiveness and cost-effectiveness. In England and Wales, discussions of the diagnostic pathway feature in the draft guidance from the National Institute for Health and Care Excellence (NICE) outlining the reasons for rejecting lecanemab and donanemab for public reimbursement [13, 25]. NICE notes that its current dementia diagnostic guideline [1] offers no guidance on diagnosing MCI due to AD [13, 25]. It moreover recognises the need, if ATTs were to be approved, for investments in further specialist diagnostic clinics to diagnose AP using PET or CSF [13]. Given the cost, time requirements and limited availability of PET/CSF [26], the biomarker-based triage in non-specialist setting would substantially reduce the wasteful use of PET/CSF resources and dementia specialist time on false positive cases. This case is supported by simulation studies that have shown blood biomarkers with complementary MMSE to substantially reduce wait times for specialist assessment [27], and more accurate non-specialist assessment strategies to be highly cost-effective versus usual (less accurate) practice [28, 29].

Similar considerations apply to the screening of eligible participants for ATT trials [14]. Around 30% of trials of disease-modifying treatments for AD target MCI or mild AD subjects [10]. However, around 30% of participants enrolled in trials of ATTs are amyloid negative [13]. A previous cost-benefit analysis estimated that the combination of plasma Aβ42 and Aβ40 and APOE4 status, achieving sensitivity of 0.88 and specificity of 0.68 for detecting AP at the Youden index cut-off, would reduce the PET cost for ATT trials by a third (from $9.2 to $6.0 million per 1,000 participants) [6]. Much greater savings can be expected from the diagnostic strategies identified in this study, given for instance that the biomarker panel plus MMSE, adjusted for covariates, achieved sensitivity of 0.910 and specificity of 0.845 for detecting MCI-PAD-AP (i.e., the trial population itself rather than just AP). Overall, we anticipate individual biomarkers or panels, combined with clinical and functional covariates, to serve as triage in streamlining specialist diagnostic work-up and ATT trial recruitment; how they may be integrated within specialist work-up is beyond the scope of this study.

In conducting analyses with PAD and MCI-PAD with or without AP as the diagnostic outcomes, an implicit assumption in this study was that biomarkers of AD pathology alone are insufficient to determine patient flow through the dementia diagnosis pathway. That is, clinical judgement of symptom presentation and severity remains a vital component of the specialist diagnostic workup and of the post-diagnostic care planning; in the absence of cognitive or functional impairment, an abnormal blood biomarker reading alone is not a good indication of clinical status, even if it performs well in detecting amyloid positivity. Thus, the current approach attempts to evaluate how the blood biomarker-based triage might be deployed to streamline a diagnostic pathway that regards AD as a clinico-pathological entity. A previous study [30] employed a similar approach to assess the comparative performance of two plasma biomarkers – (1) the amyloid probability score 2 (APS2) that combines p-tau217 and Aβ42/40 in a logistic regression to derive the probability of AP, and (2) ratio of p-tau217 and non-p-tau217 (%p-tau217) – in predicting clinical AD (clinical symptoms of AD according to clinical consensus using the International Working Group criteria [31] plus AP according to PET or CSF) relative to diagnoses by primary care physicians and secondary care dementia specialists who had no access to clinical consensus result or plasma, PET or CSF confirmation of AD pathology. Both APS2 and %p-tau217 showed significantly higher accuracy (91% accuracy for both) than physicians (61%) and specialists (73%) [30]. Further research could use the Bio-Hermes dataset to evaluate the comparative performance of biomarker panels in detecting AP alone (without clinical classification) or use an alternative dataset that contains autopsy results for AD pathology outcome (as done in e.g. [32]), .

The current approach nevertheless brings a key limitation that clinical judgement may be inaccurate, particularly for differential diagnosis of AD and non-AD subtypes of dementia. This is affirmed by the finding that 38.7% of participants with clinical judgement of PAD were amyloid negative. This in turn suggests that analyses with PAD as outcome offer limited clinical utility given that the outcome itself requires validation. It is here instructive that additions of individual biomarkers and biomarker panels generally brought significant improvements in accuracy when PAD-AP and MCI-PAD-AP were used as outcomes, but not when for PAD and MCI-PAD were used. Specifically, in the latter case, the additions of biomarkers brought no significant improvement in models that adjusted for covariates, most importantly the FAQ measure of functional impairment. This suggests that once adjusted for measures of cognitive (MMSE) and functional (FAQ) impairments, blood biomarkers offer limited additional value in predicting all-cause MCI/dementia classified by clinical symptoms alone, in contrast to their strong performance in predicting the AD subtype of MCI/dementia.

One area characterised by substantial uncertainty is whether the introduction of blood biomarkers would alleviate or exacerbate existing inequalities in AD diagnosis. Currently, systematic *screening* for dementia risk at population-level, like cancer screening, is not recommended, mainly due to the lack of effective and cost-effective treatments to prevent or slow the disease post-diagnosis [33]. In this context, additional effort may be required to ensure a high uptake of biomarker testing in underserved communities, particularly ethnic minority groups. The Bio-Hermes study has illustrated the capacity to recruit from underserved communities, specifically racial and ethnic minorities in the US [14]. The current results (Table 4) moreover show that the diagnostic accuracies of MMSE plus biomarker panel by outcome are broadly comparable between non-Hispanic White and other race/ethnicity subgroups. However, the composition of the biomarker panel differed between race/ethnicity subgroups, suggesting the need for ethnicity-tailored biomarker testing. A previous systematic review [34] identified studies where the diagnostic accuracy of individual blood biomarkers (Aβ42/40, p-tau181, p-tau217, NfL) differed by race/ethnicity and where race/ethnicity was a significant covariate in multivariate models estimating accuracy of individual biomarkers (p-tau181, p-tau231, NfL). These are complemented by findings of significant differences in blood biomarker levels across racial/ethnic subgroups (though not all studies report such difference [35]), prompting suggestions that race/ethnicity-specific reference ranges or cut-offs for biomarkers are required [36–38]. It was indeed noted that the threshold probabilities calculated by the Youden index were higher for other race/ethnicity subgroup than the non-Hispanic White subgroup across outcomes. All things equal, higher threshold implies higher specificity and lower sensitivity to reduce the rate of false positives. This in turn suggests that the other race/ethnicity subgroup have higher biomarker or risk level for a given rate of outcome. Overall, these findings of racial/ethnic differences in the optimal biomarker panel and cut-off thresholds contribute to the inequalities debate, and further research is warranted on whether the differences in biomarker performance and implementation strategy for underserved groups are mediated by natural variations in blood biomarker levels (adjusted for clinical status) and/or comorbidity profiles.

The current study employed a statistical approach to identify the best performing individual biomarker and biomarker panel for each outcome, using the Youden index to estimate the optimal cut-off for probability scores generated by multivariate logistic models. This potentially raises issues of practicality: the physician would have to order different biomarkers and track different covariates depending on the targeted outcome and base referrals on probability scores rather than individual biomarker levels. To facilitate implementation, the healthcare system commissioning the biomarker-based triage would have to specify the primary outcome to define the optimal biomarker panel and covariates (e.g., MMSE, all five blood biomarkers, age, alcohol use, APOE4 carrier status and FAQ for MCI-PAD-AP outcome; see Table A.7 in Appendix) – though these may differ by racial/ethnic subgroup. It should be noted that blood biomarkers which have received regulatory approvals already work as panels with and without probability-based cut-offs: e.g., the aforementioned APS2 approved in the UK [39] and the Fujirebio’s Lumipulse p-tau217/Aβ42 ratio approved in the US [40]. The approvals thus provide at least a preliminary indication that implementing biomarker panels in routine clinical setting would be feasible. Nevertheless, a cost-benefit analysis is warranted to evaluate the net gains of employing a complex combination of blood biomarkers, questionnaires and risk factors vis-à-vis a simplified yet still accurate strategy that combines a single biomarker like p-tau217 and MMSE. Moreover, routine implementation would be greatly strengthened by the development of a clinically integrated tool, like the QRISK3 algorithm for 10-year risk of cardiovascular disease [41, 42], which can calculate the probability score (or binary indicator of whether the score crosses the optimal cut-off) after minimal data input from clinicians.

The current study has some key strengths. First, the study evaluated the comparative performance of blood biomarkers relative to what plausibly represents current practice, namely MMSE-based cognition testing [28]. This ensures that the study results can directly inform decision-making concerning the addition of blood biomarkers to current practice. One previous study included MMSE, two further cognition tests, six blood biomarkers, and covariates (age, gender, education, BMI, and APOE4) into a decision tree model and estimated AUC of 0.935 for predicting PET-measured Aβ level [43]. However, the study did not compare the performance to models containing cognition tests alone. Second, a broad set of covariates were considered for inclusion in the regression models. Importantly, the covariates, such as alcohol/tobacco use, are readily available in general clinical settings and genetic testing for APOE4 would be feasible through blood collection. By contrast, previous studies on diagnostic and prognostic performance of blood biomarkers have generally limited the covariates to age, sex and APOE4 status [17]. The strong associations found in this study between APOE4 status and outcomes with AP suggest that APOE4 testing should be an integral part of the diagnostic pathway for ATT. The inclusion of alcohol and tobacco uses as covariates in best-fitting regression models for outcomes PAD-AP and MCI-PAD-AP is also important. It suggests that these routinely recorded lifestyle risk factors offer useful information on cognitive and functional impairment beyond age, MMSE, blood biomarkers, APOE4, and FAQ. Further research should more rigorously explore cross-sectional and longitudinal associations between tobacco and alcohol uses and AD pathology independent of clinical symptoms. For primary care physicians, this finding should motivate efforts to understand patients’ alcohol and tobacco use history, not only to inform preventive lifestyle interventions but also to improve risk stratification used in triage. Third, the study conducted analyses of NfL and GFAP which were not previously done on the Bio-Hermes cohort [14]. NfL and GFAP are non-AD-specific biomarkers for neurodegeneration and neuroinflammation, respectively [7], and the current results (Table 3) suggest that they should be combined with Aβ/tau biomarkers to detect MCI-PAD-AP. GFAP and NfL are likely associated with symptom severity, as demonstrated in a previous study [15] which found GFAP and NfL, but not p-tau181, being significantly associated with longitudinal symptom progressions in MCI and AD patients over 36 months. It is thus plausible that GFAP and NfL assist distinguishing between clinical statuses after adjusting for the AD pathology detected by the AD-specific biomarkers.

The study nevertheless has limitations. First, the same MMSE and FAQ measurements included in analyses to predict clinical outcomes had been used at participant enrolment to verify judgement on the clinical status if judgement according to the NIA-AA criteria was insufficient, though the proportion of participants for whom this was necessary was not reported [14]. This may have overestimated the predictive power of MMSE and FAQ and thereby underestimated the diagnostic value brought by the addition of blood biomarkers. That said, accuracies of MMSE and FAQ in this dataset are broadly comparable to those found in the literature: e.g., sensitivity 0.726 and specificity 0.949 at MMSE cut-off of 24 in this dataset versus 0.850 and 0.900 in the literature [18]; and sensitivity 0.681 and specificity 0.890 at FAQ cut-off of 6 in this dataset versus 0.941 and 0.751 in the literature [44]. Second, the lack of tau biomarker data obtained through PET or CSF meant that the accuracy of blood-based biomarkers to detect biological AD could not be assessed. Third, the study determined amyloid positivity based on PET or CSF measurements despite potential discordance between the two measurements. That said, the small proportion (11 of 956; 1.2%) of participants that received CSF instead of PET means that the impact of any discordance is likely to be minimal. Fourth, although the analyses adjusted for a wide range of covariates, confounding from unobserved covariates cannot be ruled out. Renal impairment, for instance, has been found to be associated with elevated plasma biomarker levels [45, 46]; however, the lack of consistent measures of renal impairment or chronic kidney disease in the Bio-Hermes dataset precluded their inclusion as covariates. Fifth, the study’s cross-sectional design meant that the capacity of blood-based biomarkers to predict longitudinal cognitive decline and prognosis could not be assessed. Sixth, we made assumptions when handling blood biomarker values above or below the upper and lower limits of detection or quantitation, though we followed biomarker-specific methods used in previous analysis of Bio-Hermes data [14]. This demonstrates how clinical implementation of biomarkers would require assay- and laboratory-specific standardisation of how values below or above limits are reported and integrated into decision algorithms. Finally, the study cohort was not split into random samples to validate in one sample the diagnostic accuracy performance estimated in another. Further research should validate in external samples the best-fit logistic regression models identified by the current study. Moreover, until cut-offs are established and full validation completed, the regression models presented here should not be interpreted as clinical prediction models ready to be implemented in clinical practice.

## Conclusions

Addition of blood biomarkers of AD pathology to MMSE and adjusted for variables readily available in general clinical settings substantially increases the accuracy of detecting amyloid-positive MCI and dementia cases. This could potentially support an efficient and equitable diagnostic pathway that improves access to treatments targeting AD pathology. Further research should fully assess the health, economic, and social benefits of implementing such diagnostic pathway and validate the diagnostic and prognostic performance of biomarkers in different populations.

## Supplementary Information

Supplementary Material 1

## Data Availability

Data was released by Bio-Hermes through the AD Workbench hosted by AD Data Initiative. Individual participant data is available through the same platform pending agreement from AD Data Initiative. Study protocol, data dictionary and R scripts for the variables and analyses used in this manuscript is available from the corresponding author upon reasonable request upon manuscript publication.

## Abbreviations

Aβ: Beta-amyloid
AD: Alzheimer’s disease
AIC: Akaike information criterion
ANOVA: Analysis of variance
AP: Amyloid positivity
ATT: Amyloid-targeting treatment
AUC: Area under the receiver operating characteristic curve
BIC: Bayesian information criterion
CSF: Cerebrospinal fluid
FAQ: Functional Activity Questionnaire
GFAP: Glial fibrillary acidic protein
MCI: Mild cognitive impairment
MMSE: Mini-Mental State Examination
NfL: Neurofilament light
NIA-AA: National Institute on Aging and Alzheimer’s Association
NICE: National Institute for Health and Care Excellence
p-tau: Phosphorylated tau
PAD: Probable Alzheimer’s disease
PET: Positron emission tomography
RAVLT: Rey Auditory Verbal Learning Test
ROC: Receiver operating characteristic
SD: Standard deviation
STARD: Standards for Reporting of Diagnostic Accuracy Studies
