# Supplementary material for: Blood biomarkers to improve dementia diagnostic accuracy: a cross-sectional analysis
Source: BMC Geriatr. 2026 May 1;26:888. doi: 10.1186/s12877-026-07431-9 (PMC13321689; doi:10.1186/s12877-026-07431-9)
Supplement: Supplementary file 1 — Supplementary Material 1 [file 12877_2026_7431_MOESM1_ESM.docx]

# Blood biomarkers to improve diagnostic accuracy in dementia: a cross-sectional analysis

**APPENDIX**

**Authors:**

Joseph Kwon, PhD,*^1^ [joseph.kwon@phc.ox.ac.uk](mailto:joseph.kwon@phc.ox.ac.uk); ORCID 0000-0002-2860-7280

Megan Kirk Chang, PhD,^1^ [megan.kirkchang@phc.ox.ac.uk](mailto:megan.kirkchang@phc.ox.ac.uk); ORCID 0000-0002-2069-2177

Adam Gordon-Boyle, BA,^2^ [adam.gordon-boyle@worc.ox.ac.uk](mailto:adam.gordon-boyle@worc.ox.ac.uk); ORCID 0009-0003-4847-3210

Sam Creavin, PhD,^3^ [sam.creavin@bristol.ac.uk](mailto:sam.creavin@bristol.ac.uk); ORCID 0000-0002-6772-7111

Lynne Hughes, PhD,^4^ [lhughes@globalalzplatform.org](mailto:lhughes@globalalzplatform.org)

Vanessa Raymont, PhD,^5^ [vanessa.raymont@psych.ox.ac.uk](mailto:vanessa.raymont@psych.ox.ac.uk); ORCID 0000-0001-8238-4279

Kamaldeep Bhui, PhD,^1,5^ [kam.bhui@psych.ox.ac.uk](mailto:kam.bhui@psych.ox.ac.uk); ORCID 0000-0002-9205-2144

Apostolos Tsiachristas, PhD,^1,5^ [apostolos.tsiachristas@phc.ox.ac.uk](mailto:apostolos.tsiachristas@phc.ox.ac.uk); ORCID 0000-0002-4662-8915

* Corresponding author

^1^ Nuffield Department of Primary Care Health Sciences, University of Oxford, Radcliffe Primary Care Building, Radcliffe Observatory Quarter, Woodstock Road, Oxford, England, OX2 6GG

^2^ Medical Sciences Division, University of Oxford, John Radcliffe Hospital, Headley Way, Headington, Oxford, OX3 9DU

^3^ Centre for Academic Primary Care, Bristol Medical School, University of Bristol, 39 Whatley Road, Clifton, Bristol, BS8 2PS

^4^ Global Alzheimer's Platform Foundation, 4315 50th St. NW Ste 100 Unit 2623 Washington, DC 20016

^5^ Department of Psychiatry, University of Oxford, Warneford Hospital, Oxford, OX3 7JX

Table of Contents

[Blood biomarkers to improve diagnostic accuracy in dementia: a cross-sectional predictive analysis 1](#_Toc202618063)

[STARD reporting checklist 3](#_Toc202618064)

[Data preparation 5](#_Toc202618065)

[Sample characteristics 6](#_Toc202618066)

[Diagnostic accuracy of MMSE plus individual biomarkers 7](#_Toc202618067)

[Diagnostic accuracy of MMSE plus biomarker panels 16](#_Toc202618068)

[Subgroup analyses 17](#_Toc202618069)

[References 21](#_Toc202618070)

# STARD reporting checklist

|  | **Section & Topic** | **No** | **Item** | **Reported on page #** |
| --- | --- | --- | --- | --- |
|  |  |  |  |  |
|  | **TITLE OR ABSTRACT** |  |  |  |
|  |  | **1** | Identification as a study of diagnostic accuracy using at least one measure of accuracy (such as sensitivity, specificity, predictive values, or AUC) | Title and Abstract |
|  | **ABSTRACT** |  |  |  |
|  |  | **2** | Structured summary of study design, methods, results, and conclusions  (for specific guidance, see STARD for Abstracts) | Abstract |
|  | **INTRODUCTION** |  |  |  |
|  |  | **3** | Scientific and clinical background, including the intended use and clinical role of the index test | First four paragraphs of Background |
|  |  | **4** | Study objectives and hypotheses | Last paragraph of Background |
|  | **METHODS** |  |  |  |
|  | *Study design* | **5** | Whether data collection was planned before the index test and reference standard were performed (prospective study) or after (retrospective study) | Not applicable |
|  | *Participants* | **6** | Eligibility criteria | Given reference to BioHermes study (Mohs et al., 2024) |
|  |  | **7** | On what basis potentially eligible participants were identified  (such as symptoms, results from previous tests, inclusion in registry) |  |
|  |  | **8** | Where and when potentially eligible participants were identified (setting, location and dates) |  |
|  |  | **9** | Whether participants formed a consecutive, random or convenience series |  |
|  | *Test methods* | **10a** | Index test, in sufficient detail to allow replication | ‘Diagnostic outcomes’ and ‘Diagnostic tests and covariates’ |
|  |  | **10b** | Reference standard, in sufficient detail to allow replication |  |
|  |  | **11** | Rationale for choosing the reference standard (if alternatives exist) |  |
|  |  | **12a** | Definition of and rationale for test positivity cut-offs or result categories  of the index test, distinguishing pre-specified from exploratory | Youden index used: see ‘Statistical analyses’ |
|  |  | **12b** | Definition of and rationale for test positivity cut-offs or result categories  of the reference standard, distinguishing pre-specified from exploratory |  |
|  |  | **13a** | Whether clinical information and reference standard results were available  to the performers/readers of the index test | Not applicable |
|  |  | **13b** | Whether clinical information and index test results were available  to the assessors of the reference standard | Not applicable |
|  | *Analysis* | **14** | Methods for estimating or comparing measures of diagnostic accuracy | ‘Statistical analyses’ |
|  |  | **15** | How indeterminate index test or reference standard results were handled | Not applicable |
|  |  | **16** | How missing data on the index test and reference standard were handled | ‘Diagnostic tests and covariates’ |
|  |  | **17** | Any analyses of variability in diagnostic accuracy, distinguishing pre-specified from exploratory | ‘Subgroup analyses’ |
|  |  | **18** | Intended sample size and how it was determined | Given reference to BioHermes study (Mohs et al., 2024) |
|  | **RESULTS** |  |  |  |
|  | *Participants* | **19** | Flow of participants, using a diagram | Given reference to BioHermes study (Mohs et al., 2024) |
|  |  | **20** | Baseline demographic and clinical characteristics of participants | Tables 1 and A2 |
|  |  | **21a** | Distribution of severity of disease in those with the target condition | Tables 1 and A2 |
|  |  | **21b** | Distribution of alternative diagnoses in those without the target condition | Tables 1 and A2 |
|  |  | **22** | Time interval and any clinical interventions between index test and reference standard | Not applicable |
|  | *Test results* | **23** | Cross tabulation of the index test results (or their distribution)  by the results of the reference standard | Not reported |
|  |  | **24** | Estimates of diagnostic accuracy and their precision (such as 95% confidence intervals) | Tables 2-4 and A3-A12 |
|  |  | **25** | Any adverse events from performing the index test or the reference standard | Not applicable |
|  | **DISCUSSION** |  |  |  |
|  |  | **26** | Study limitations, including sources of potential bias, statistical uncertainty, and generalisability | Last paragraph of Discussion |
|  |  | **27** | Implications for practice, including the intended use and clinical role of the index test | 2^nd^ to 5^th^ paragraphs of Discussion |
|  | **OTHER INFORMATION** |  |  |  |
|  |  | **28** | Registration number and name of registry | Not applicable |
|  |  | **29** | Where the full study protocol can be accessed | ‘Declaration of interests’ |
|  |  | **30** | Sources of funding and other support; role of funders | ‘Role of the funding source’ and ‘Declaration of interests’ |
|  |  |  |  |  |

# Data preparation

| **Table A.1** Data imputation and outlier removal | | |
| --- | --- | --- |
| **Variable** | **Processing** | **# of observations removed** |
| p-tau181 | Value marked as “<0.1” (n=1) set to 0 pg/mL. This was done in previous publication on Bio-Hermes data [1]. | 0 |
| p-tau217 | For values below the lower limit of quantitation (n=175), random values between 0 and 0.131 U/mL (minimum specified value) were assigned. For values above the upper limit of quantitation (n=7), the maximum specified value of 1.39 U/mL was assigned. These steps were used in previous publication on Bio-Hermes data [1].  Mean = 0.247 U/mL, SD = 0.224. Hence 1.39 U/mL ceiling is around 5 SDs above mean value. A similar rule was applied to other biomarkers: values that are 6 SDs greater or smaller than the sample mean were treated as outliers and removed. Note that for normally distributed variables, outliers are defined as those 3 SDs greater or smaller than the mean [2]. | 0 |
| Aβ42 | Outliers defined as six standard deviations or more above the mean removed.  Mean = 53.9 pg/mL, SD = 54.0; 10 values between 437.6 and 733.7 pg/mL removed due to being more than 6 SDs greater than mean | 10 |
| GFAP | Outliers defined as six standard deviations or more above the mean removed  Mean = 112.6 pg/mL, SD = 64.6; 2 values between 634.0 and 712.7 pg/mL removed due to being more than 6 SDs greater than mean | 2 |
| NfL | Outliers defined as six standard deviations or more above the mean removed  Mean = 3.528 pg/mL, SD = 2.757; 4 values between 20.41 and 43.96 pg/mL removed due to being more than 6 SDs greater than mean | 4 |
| Blood pressure | High blood pressure if systolic blood pressure (SBP) was above or equal to 140 mmHg or diastolic blood pressure (DBP) was above or equal to 90 mmHg [3]. Removed SBP values below 20 mmHg (n=4) as outliers or errors and DBP values above 800 mmHg (n=2). | 6 |
| Weight | Obese if body mass index (BMI) ≥30; overweight if 25≤ BMI <30. Removed BMI values above 4,000 (n=2) | 2 |
| Depression | Depression if the Geriatric Depression Scale (GDS, range 0-15) score ≥5 [4] or if participant has medical record of ongoing depression or major depression | 0 |
| **Abbreviation:** SD: standard deviation | | |

# Sample characteristics

| **Table A.2** Sample characteristics by clinical judgement | | | | |
| --- | --- | --- | --- | --- |
|  | **CN (n=394)** | **MCI (n=286)** | **PAD (n=248)** | ***P*-value** |
| Amyloid positive, n (%) | 83 (21.1) | 103 (36.0) | 152 (61.3) | **<0.001** |
| Age (years), mean (SD) | 70.3 (6.4) | 72.2 (6.8) | 74.4 (6.1) | **<0.001** |
| Female, n (%) | 241 (61.2) | 156 (54.5) | 131 (52.8) | 0.072 |
| Non-Hispanic White, n (%) | 320 (81.2) | 221 (77.3) | 169 (68.1) | **<0.001** |
| High blood pressure,^a^ n (%) | 132 (33.5) | 91 (31.8) | 107 (43.1) | **0.013** |
| Overweight/ obese,^b^ n (%) | 278 (70.6) | 182 (63.6) | 145 (58.5) | **0.006** |
| Alcohol use, n (%) | 293 (74.4) | 208 (72.7) | 157 (63.3) | **0.008** |
| Tobacco use, n (%) | 143 (36.3) | 122 (42.7) | 91 (36.7) | 0.198 |
| Depression,^c^ n (%) | 102 (25.9) | 90 (31.5) | 90 (36.3) | **0.018** |
| APOE4 carrier, n (%) | 128 (32.5) | 108 (37.8) | 113 (45.6) | **0.004** |
| Aβ42/40,^d^ mean (SD) | 9.844 (0.968) | 9.635 (0.980) | 9.578 (0.985) | **0.001** |
| p-tau181 (pg/mL), mean (SD) | 0.900 (0.380) | 1.060 (0.555) | 1.387 (0.656) | **<0.001** |
| p-tau217 (U/mL),^d^ mean (SD) | 17.0 (9.8) | 23.1 (19.9) | 38.6 (30.5) | **<0.001** |
| GFAP (pg/mL), mean (SD) | 94.9 (47.1) | 112.6 (60.8) | 137.4 (65.8) | **<0.001** |
| NfL (pg/mL), mean (SD) | 2.839 (1.540) | 3.549 (2.148) | 4.256 (2.515) | **<0.001** |
| MMSE total, mean (SD) | 28.4 (1.5) | 27.2 (1.9) | 23.3 (2.5) | **<0.001** |
| FAQ, mean (SD) | 0.7 (1.5) | 3.6 (4.4) | 9.1 (6.4) | **<0.001** |
| ^a^ High if DBP ≥90 mmHg or SBP ≥140 mmHg.  ^b^ Obese if BMI ≥30; overweight if 25≤ BMI <30.  ^c^ Depressed if Geriatric Depression Scale (GDS) ≥5 or has ongoing (major) depression according to medical history.  ^d^ Multiplied by 100 for ease of interpreting logistic regression coefficients.  **Abbreviation:** AP: amyloid positivity; APOE4: apolipoprotein E allele 4; DBP: diastolic blood pressure; FAQ: Functional Activities Questionnaire; GFAP: glial fibrillary acidic protein; MCI: mild cognitive impairment; MMSE: Mini-Mental State Examination; NfL: neurofilament light; PAD: probable Alzheimer’s disease; SBP: systolic blood pressure; SD: standard deviation. | | | | |

# Diagnostic accuracy of MMSE plus individual biomarkers

| **Table A.3** Accuracy of MMSE plus individual blood biomarkers to detect PAD. | | | | | | | | | | | | |
| --- | --- | --- | --- | --- | --- | --- | --- | --- | --- | --- | --- | --- |
|  | **(1) MMSE** | **(2) MMSE, adjusted** | **(3) MMSE + Aβ42/40^a^** | **(4) MMSE + Aβ42/40^a^, adjusted** | **(5) MMSE + p-tau181** | **(6) MMSE + p-tau181, adjusted** | **(7) MMSE + p-tau217^a^** | **(8) MMSE + p-tau217^a^, adjusted** | **(9) MMSE + GFAP** | **(10) MMSE + GFAP, adjusted** | **(11) MMSE + NfL** | **(12) MMSE + NfL, adjusted** |
| **Logistic regression odds ratios (bold if *P*<0.05). Sample n = 928** | | | | | | | | | | | | |
| Intercept | **1.628 e+9**  **(*P*<0.001)** | **9.102 e+7**  **(*P*<0.001)** | **1.502 e+9**  **(*P*<0.001)** | **1.739 e+5**  **(*P*<0.001)** | **3.421 e+8**  **(*P*<0.001)** | **4.581 e+7**  **(*P*<0.001)** | **3.962 e+8**  **(*P*<0.001)** | **3.696 e+7**  **(*P*<0.001)** | **6.815 e+8**  **(*P*<0.001)** | **7.865 e+7**  **(*P*<0.001)** | **7.825 e+8**  **(*P*<0.001)** | **5.763 e+6**  **(*P*<0.001)** |
| MMSE | **0.422**  **(*P*<0.001)** | **0.454**  **(*P*<0.001)** | **0.422**  **(*P*<0.001)** | **0.449**  **(*P*<0.001)** | **0.433**  **(*P*<0.001)** | **0.459**  **(*P*<0.001)** | **0.436**  **(*P*<0.001)** | **0.464**  **(*P*<0.001)** | **0.429**  **(*P*<0.001)** | **0.455**  **(*P*<0.001)** | **0.426**  **(*P*<0.001)** | **0.453**  **(*P*<0.001)** |
| Blood biomarker | - | - | 1.009  (*P*=0.934) | **1.382**  **(*P*=0.012)** | **2.258**  **(*P*<0.001)** | 1.459  (*P*=0.076) | **1.023**  **(*P*<0.001)** | **1.014**  **(*P*=0.007)** | **1.004**  **(*P*=0.018)** | 1.001  (*P*=0.693) | **1.148**  **(*P*=0.008)** | 0.947  (*P*=0.403) |
| Age | - | Excluded^b^ | - | **1.047**  **(*P*=0.022)** | - | Excluded | - | Excluded | - | Excluded | - | **1.042**  **(*P*=0.047)** |
| Female | - | Excluded | - | Excluded | - | Excluded | - | Excluded | - | Excluded | - | Excluded |
| Non-Hispanic White | - | Excluded | - | Excluded | - | Excluded | - | Excluded | - | Excluded | - | Excluded |
| Overweight/ obese | - | Excluded | - | Excluded | - | Excluded | - | Excluded | - | Excluded | - | Excluded |
| Alcohol use | - | Excluded | - | Excluded | - | Excluded | - | Excluded | - | Excluded | - | Excluded |
| Tobacco use | - | Excluded | - | Excluded | - | Excluded | - | Excluded | - | Excluded | - | Excluded |
| High blood pressure | - | Excluded | - | Excluded | - | Excluded | - | Excluded | - | Excluded | - | Excluded |
| Depression | - | Excluded | - | Excluded | - | Excluded | - | Excluded | - | Excluded | - | Excluded |
| APOE4 carrier | - | - | - | Excluded | - | Excluded | - | Excluded | - | Excluded | - | Excluded |
| FAQ | **-** | **1.238**  **(*P*<0.001)** | - | **1.247**  **(*P*<0.001)** | - | **1.225**  **(*P*<0.001)** | - | **1.224**  **(*P*<0.001)** | - | **1.236**  **(*P*<0.001)** | - | **1.240**  **(*P*<0.001)** |
| AIC/n | 0.582 | 0.487 | 0.584 | 0.481 | 0.564 | 0.486 | 0.560 | 0.481 | 0.578 | 0.489 | 0.576 | 0.487 |
| BIC/n | 0.592 | 0.503 | 0.599 | 0.507 | 0.580 | 0.507 | 0.576 | 0.502 | 0.593 | 0.510 | 0.592 | 0.513 |
| **Test accuracy** | | | | | | | | | | | | |
| AUC | 0.920 | 0.950 | 0.919 | 0.952 | 0.925 | 0.951 | 0.927 | 0.952 | 0.924 | 0.950 | 0.925 | 0.952 |
| AUC 95% CI | 0.898-0.943 | 0.934-0.966 | 0.897-0.942 | 0.937-0.968 | 0.904-0.947 | 0.935-0.967 | 0.905-0.949 | 0.936-0.968 | 0.903-0.945 | 0.935-0.966 | 0.904-0.947 | 0.936-0.967 |
| Threshold probability | 25.5^c^ | 0.312 | 0.235 | 0.369 | 0.242 | 0.294 | 0.241 | 0.249 | 0.257 | 0.300 | 0.236 | 0.371 |
| Sensitivity | 0.827 | 0.891 | 0.835 | 0.867 | 0.875 | 0.895 | 0.879 | 0.911 | 0.847 | 0.891 | 0.867 | 0.863 |
| Specificity | 0.893 | 0.909 | 0.885 | 0.935 | 0.865 | 0.904 | 0.868 | 0.887 | 0.875 | 0.907 | 0.863 | 0.929 |
| ^a^ Multiplied by 100 for ease of interpreting logistic regression coefficients.  ^b^ Excluded because the covariate was not significantly associated with outcome at 95% significance level.  ^c^ Value of MMSE rather than probability estimated by logistic regression.  **Abbreviation:** AIC: Akaike information criterion; APOE4: apolipoprotein E allele 4; AUC: area under the curve; BIC: Bayesian information criterion; CI: confidence interval; FAQ: Functional Activities Questionnaire; GFAP: glial fibrillary acidic protein; MMSE: Mini-Mental State Examination; NfL: neurofilament light; PAD: probable Alzheimer’s disease. | | | | | | | | | | | | |
| **Table A.4** Accuracy of MMSE plus individual blood biomarkers to detect MCI-PAD | | | | | | | | | | | | |
|  | **(1) MMSE** | **(2) MMSE, adjusted** | **(3) MMSE + Aβ42/40^a^** | **(4) MMSE + Aβ42/40^a^, adjusted** | **(5) MMSE + p-tau181** | **(6) MMSE + p-tau181, adjusted** | **(7) MMSE + p-tau217^a^** | **(8) MMSE + p-tau217^a^, adjusted** | **(9) MMSE + GFAP** | **(10) MMSE + GFAP, adjusted** | **(11) MMSE + NfL** | **(12) MMSE + NfL, adjusted** |
| **Logistic regression odds ratios (bold if *P*<0.05). Sample n = 928** | | | | | | | | | | | | |
| Intercept | **8.901 e+6**  **(*P*<0.001)** | **1.130 e+5**  **(*P*<0.001)** | **7.156 e+7**  **(*P*<0.001)** | **1.771 e+5**  **(*P*<0.001)** | **1.914 e+6**  **(*P*<0.001)** | **6.722 e+4**  **(*P*<0.001)** | **1.627 e+6**  **(*P*<0.001)** | **5.996 e+4**  **(*P*<0.001)** | **2.678 e+6**  **(*P*<0.001)** | **8.843 e+4**  **(*P*<0.001)** | **2.539 e+6**  **(*P*<0.001)** | **7.738 e+4**  **(*P*<0.001)** |
| MMSE | **0.562**  **(*P*<0.001)** | **0.638**  **(*P*<0.001)** | **0.561**  **(*P*<0.001)** | **0.637**  **(*P*<0.001)** | **0.576**  **(*P*<0.001)** | **0.641**  **(*P*<0.001)** | **0.584**  **(*P*<0.001)** | **0.645**  **(*P*<0.001)** | **0.574**  **(*P*<0.001)** | **0.640**  **(*P*<0.001)** | **0.573**  **(*P*<0.001)** | **0.639**  **(*P*<0.001)** |
| Blood biomarker | - | - | **0.809**  **(*P*=0.010)** | 0.958  (*P*=0.639) | **2.311**  **(*P*<0.001)** | **1.532**  **(*P*=0.036)** | **1.031**  **(*P*<0.001)** | **1.018**  **(*P*=0.011)** | **1.006**  **(*P*<0.001)** | 1.002  (*P*=0.366) | **1.248**  **(*P*<0.001)** | **1.117**  **(*P*=0.038)** |
| Age | **-** | Excluded^b^ | - | Excluded | - | Excluded | - | Excluded | - | Excluded | - | Excluded |
| Female | **-** | Excluded | - | Excluded | - | Excluded | - | Excluded | - | Excluded | - | Excluded |
| Non-Hispanic White | - | Excluded | - | Excluded | - | Excluded | - | Excluded | - | Excluded | - | Excluded |
| Overweight/ obese | **-** | Excluded | - | Excluded | - | Excluded | - | Excluded | - | Excluded | - | Excluded |
| Alcohol use | - | Excluded | - | Excluded | - | Excluded | - | Excluded | - | Excluded | - | Excluded |
| Tobacco use | - | Excluded | - | Excluded | - | Excluded | - | Excluded | - | Excluded | - | Excluded |
| High blood pressure | - | Excluded | - | Excluded | - | Excluded | - | Excluded | - | Excluded | - | Excluded |
| Depression | **-** | Excluded | - | Excluded | - | Excluded | - | Excluded | - | Excluded | - | Excluded |
| APOE4 carrier | - | - | - | Excluded | - | Excluded | - | Excluded | - | Excluded | - | Excluded |
| FAQ | - | **1.563**  **(*P*<0.001)** | - | **1.558**  **(*P*<0.001)** | - | **1.539**  **(*P*<0.001)** | - | **1.526**  **(*P*<0.001)** | - | **1.550**  **(*P*<0.001)** | - | **1.541**  **(*P*<0.001)** |
| AIC/n | 1.024 | 0.837 | 1.019 | 0.839 | 1.001 | 0.835 | 0.994 | 0.831 | 1.011 | 0.838 | 1.001 | 0.834 |
| BIC/n | 1.035 | 0.853 | 1.035 | 0.860 | 1.017 | 0.855 | 1.009 | 0.852 | 1.027 | 0.859 | 1.017 | 0.855 |
| **Test accuracy** | | | | | | | | | | | | |
| AUC | 0.807 | 0.876 | 0.813 | 0.878 | 0.819 | 0.881 | 0.819 | 0.879 | 0.815 | 0.879 | 0.820 | 0.881 |
| AUC 95% CI | 0.780-0.834 | 0.854-0.898 | 0.787-0.840 | 0.857-0.900 | 0.792-0.845 | 0.859-0.902 | 0.792-0.845 | 0.857-0.901 | 0.789-0.842 | 0.857-0.900 | 0.794-0.847 | 0.859-0.902 |
| Threshold probability | 26.5^c^ | 0.596 | 0.638 | 0.588 | 0.628 | 0.511 | 0.658 | 0.625 | 0.649 | 0.584 | 0.668 | 0.584 |
| Sensitivity | 0.605 | 0.734 | 0.646 | 0.751 | 0.657 | 0.787 | 0.654 | 0.713 | 0.644 | 0.743 | 0.644 | 0.738 |
| Specificity | 0.886 | 0.878 | 0.860 | 0.871 | 0.858 | 0.838 | 0.886 | 0.911 | 0.871 | 0.876 | 0.886 | 0.891 |
| ^a^ Multiplied by 100 for ease of interpreting logistic regression coefficients.  ^b^ Excluded because the covariate was not significantly associated with outcome at 95% significance level.  ^c^ Value of MMSE rather than probability estimated by logistic regression.  **Abbreviation:** AIC: Akaike information criterion; APOE4: apolipoprotein E allele 4; AUC: area under the curve; BIC: Bayesian information criterion; CI: confidence interval; FAQ: Functional Activities Questionnaire; GFAP: glial fibrillary acidic protein; MCI: mild cognitive impairment; MMSE: Mini-Mental State Examination; NfL: neurofilament light; PAD: probable Alzheimer’s disease. | | | | | | | | | | | | |

| **eTable 5** Accuracy of MMSE plus individual blood biomarkers to detect PAD with AP | | | | | | | | | | | | |
| --- | --- | --- | --- | --- | --- | --- | --- | --- | --- | --- | --- | --- |
|  | **(1) MMSE** | **(2) MMSE, adjusted** | **(3) MMSE + Aβ42/40^a^** | **(4) MMSE + Aβ42/40^a^, adjusted** | **(5) MMSE + p-tau181** | **(6) MMSE + p-tau181, adjusted** | **(7) MMSE + p-tau217^a^** | **(8) MMSE + p-tau217^a^, adjusted** | **(9) MMSE + GFAP** | **(10) MMSE + GFAP, adjusted** | **(11) MMSE + NfL** | **(12) MMSE + NfL, adjusted** |
| **Logistic regression odds ratios (bold if *P*<0.05). Sample n = 928** | | | | | | | | | | | | |
| Intercept | **2.542 e+6**  **(*P*<0.001)** | **9.832 e+3**  **(*P*<0.001)** | **3.030 e+10**  **(*P*<0.001)** | **9.464 e+5**  **(*P*<0.001)** | **1.787 e+5**  **(*P*<0.001)** | **4.013 e+2**  **(*P*=0.007)** | **2.543 e+5**  **(*P*<0.001)** | **1.723 e+2**  **(*P*=0.024)** | **3.549 e+5**  **(*P*<0.001)** | **1.559 e+3**  **(*P*<0.001)** | **8.077 e+5**  **(*P*<0.001)** | **1.042 e+3**  **(*P*=0.001)** |
| MMSE | **0.522**  **(*P*<0.001)** | **0.550**  **(*P*<0.001)** | **0.510**  **(*P*<0.001)** | **0.530**  **(*P*<0.001)** | **0.532**  **(*P*<0.001)** | **0.532**  **(*P*<0.001)** | **0.543**  **(*P*<0.001)** | **0.552**  **(*P*<0.001)** | **0.531**  **(*P*<0.001)** | **0.540**  **(*P*<0.001)** | **0.529**  **(*P*<0.001)** | **0.537**  **(*P*<0.001)** |
| Blood biomarker | - | - | **0.394**  **(*P*<0.001)** | **0.554**  **(*P*<0.001)** | **5.426**  **(*P*<0.001)** | **4.269**  **(*P*<0.001)** | **1.042**  **(*P*<0.001)** | **1.036**  **(*P*<0.001)** | **1.012**  **(*P*<0.001)** | **1.010**  **(*P*<0.001)** | **1.234**  **(*P*<0.001)** | **1.159**  **(*P*=0.018)** |
| Age | **-** | **1.044**  **(*P*=0.032)** | - | **1.063**  **(*P*=0.007)** | - | **1.065**  **(*P*=0.007)** | - | **1.074**  **(*P=*0.003)** | - | **1.049**  **(*P=*0.039)** | - | **1.066**  **(*P*=0.004)** |
| Female | - | Excluded^b^ | - | Excluded | - | Excluded | - | Excluded | - | Excluded | - | Excluded |
| Non-Hispanic White | **-** | **2.063**  **(*P*=0.013)** | - | Excluded | - | Excluded | - | Excluded | - | Excluded | - | Excluded |
| Overweight/ obese | - | Excluded | - | Excluded | - | Excluded | - | Excluded | - | Excluded | - | Excluded |
| Alcohol use | - | Excluded | - | Excluded | - | Excluded | - | Excluded | - | Excluded | - | Excluded |
| Tobacco use | - | Excluded | - | **1.881**  **(*P*=0.022)** | - | **2.064**  **(*P*=0.012)** | - | **2.173**  **(*P*=0.008)** | - | **2.015**  **(*P*=0.013)** | - | **1.817**  **(*P*=0.028)** |
| High blood pressure | - | Excluded | - | Excluded | - | Excluded | - | Excluded | - | Excluded | - | Excluded |
| Depression | - | Excluded | - | Excluded | - | Excluded | - | Excluded | - | Excluded | - | Excluded |
| APOE4 carrier | - | - | - | **5.427**  **(*P*<0.001)** | - | **6.461**  **(*P*<0.001)** | - | **5.640**  **(*P*<0.001)** | - | **6.418**  **(*P*<0.001)** | - | **7.358**  **(*P*<0.001)** |
| FAQ | - | **1.104**  **(*P*<0.001)** | - | **1.106**  **(*P*<0.001)** | - | **1.089**  **(*P*<0.001)** | - | **1.105**  **(*P*<0.001)** | - | **1.103**  **(*P*<0.001)** | - | **1.101**  **(*P*<0.001)** |
| AIC/n | 0.547 | 0.505 | 0.498 | 0.436 | 0.467 | 0.403 | 0.466 | 0.400 | 0.501 | 0.429 | 0.530 | 0.445 |
| BIC/n | 0.557 | 0.531 | 0.514 | 0.472 | 0.483 | 0.440 | 0.482 | 0.437 | 0.516 | 0.466 | 0.546 | 0.481 |
| **Test accuracy** | | | | | | | | | | | | |
| AUC | 0.903 | 0.929 | 0.922 | 0.950 | 0.933 | 0.956 | 0.934 | 0.958 | 0.923 | 0.953 | 0.915 | 0.949 |
| AUC 95% CI | 0.877-0.928 | 0.910-0.948 | 0.900-0.944 | 0.935-0.964 | 0.911-0.954 | 0.943-0.970 | 0.913-0.956 | 0.945-0.972 | 0.902-0.944 | 0.939-0.966 | 0.892-0.937 | 0.935-0.963 |
| Threshold probability | 25.5^c^ | 0.156 | 0.166 | 0.077 | 0.115 | 0.142 | 0.100 | 0.196 | 0.131 | 0.169 | 0.114 | 0.142 |
| Sensitivity | 0.855 | 0.888 | 0.868 | 0.974 | 0.921 | 0.921 | 0.941 | 0.894 | 0.888 | 0.901 | 0.901 | 0.914 |
| Specificity | 0.809 | 0.862 | 0.853 | 0.782 | 0.820 | 0.862 | 0.798 | 0.903 | 0.805 | 0.876 | 0.784 | 0.847 |
| ^a^ Multiplied by 100 for ease of interpreting logistic regression coefficients.  ^b^ Excluded because the covariate was not significantly associated with outcome at 95% significance level.  ^c^ Value of MMSE rather than probability estimated by logistic regression.  **Abbreviation:** AIC: Akaike information criterion; AP: amyloid positivity; APOE4: apolipoprotein E allele 4; AUC: area under the curve; BIC: Bayesian information criterion; CI: confidence interval; FAQ: Functional Activities Questionnaire; GFAP: glial fibrillary acidic protein; MMSE: Mini-Mental State Examination; NfL: neurofilament light; PAD: probable Alzheimer’s disease. | | | | | | | | | | | | |

| **eTable 6** Accuracy of MMSE plus individual blood biomarkers to detect MCI-PAD with AP | | | | | | | | | | | | |
| --- | --- | --- | --- | --- | --- | --- | --- | --- | --- | --- | --- | --- |
|  | **(1) MMSE** | **(2) MMSE, adjusted** | **(3) MMSE + Aβ42/40^a^** | **(4) MMSE + Aβ42/40^a^, adjusted** | **(5) MMSE + p-tau181** | **(6) MMSE + p-tau181, adjusted** | **(7) MMSE + p-tau217^a^** | **(8) MMSE + p-tau217^a^, adjusted** | **(9) MMSE + GFAP** | **(10) MMSE + GFAP, adjusted** | **(11) MMSE + NfL** | **(12) MMSE + NfL, adjusted** |
| **Logistic regression odds ratios (bold if *P*<0.05). Sample n = 928** | | | | | | | | | | | | |
| Intercept | **1.125 e+4**  **(*P*<0.001)** | 4.252  (*P*=0.308) | **3.230 e+10**  **(*P*<0.001)** | **6.003 e+5**  **(*P*<0.001)** | **2.347 e+2**  **(*P*<0.001)** | 0.188  (*P*=0.334) | **1.881 e+2**  **(*P*<0.001)** | **0.024**  **(*P*=0.039)** | **5.443 e+2**  **(*P*<0.001)** | 1.855  (*P*=0.719) | **3.126 e+3**  **(*P*<0.001)** | 0.516  (*P*=0.681) |
| MMSE | **0.674**  **(*P*<0.001)** | **0.736**  **(*P*<0.001)** | **0.639**  **(*P*<0.001)** | **0.683**  **(*P*<0.001)** | **0.707**  **(*P*<0.001)** | **0.720**  **(*P*<0.001)** | **0.734**  **(*P*<0.001)** | **0.767**  **(*P*<0.001)** | **0.701**  **(*P*<0.001)** | **0.715**  **(*P*<0.001)** | **0.689**  **(*P*<0.001)** | **0.712**  **(*P*<0.001)** |
| Blood biomarker | - | - | **0.241**  **(*P*<0.001)** | **0.317**  **(*P*<0.001)** | **9.748**  **(*P*<0.001)** | **6.624**  **(*P*<0.001)** | **1.077**  **(*P*<0.001)** | **1.060**  **(*P*<0.001)** | **1.017**  **(*P*<0.001)** | **1.015**  **(*P*<0.001)** | **1.223**  **(*P*<0.001)** | **1.113**  **(*P*=0.031)** |
| Age | **-** | **1.068**  **(*P*<0.001)** | - | **1.069**  **(*P*<0.001)** | - | **1.077**  **(*P*<0.001)** | - | **1.090**  **(*P*<0.001)** | - | **1.056**  **(*P*=0.003)** | - | **1.093**  **(*P*=0.003)** |
| Female | - | Excluded^b^ | - | Excluded | - | Excluded | - | Excluded | - | **0.621**  **(*P*=0.025)** | - | Excluded |
| Non-Hispanic White | **-** | Excluded | - | Excluded | - | Excluded | - | Excluded | - | Excluded | - | Excluded |
| Overweight/ obese | **-** | Excluded | - | Excluded | - | Excluded | - | Excluded | - | Excluded | - | Excluded |
| Alcohol use | **-** | **1.938**  **(*P*=0.001)** | - | **1.870**  **(*P*=0.008)** | - | **2.018**  **(*P*=0.004)** | - | **1.963**  **(*P*=0.006)** | - | **1.882**  **(*P*=0.007)** | - | **1.891**  **(*P*=0.004)** |
| Tobacco use | - | Excluded | - | Excluded | - | Excluded | - | Excluded | - | Excluded | - | Excluded |
| High blood pressure | **-** | **0.596**  **(*P*=0.007)** | - | Excluded | - | **0.582**  **(*P*=0.017)** | - | Excluded | - | **0.612**  **(*P*=0.025)** | - | **0.557**  **(*P*=0.005)** |
| Depression | - | Excluded | - | Excluded | - | Excluded | - | Excluded | - | Excluded | - | Excluded |
| APOE4 carrier | - | - | - | **5.562**  **(*P*<0.001)** | - | **7.252**  **(*P*<0.001)** | - | **6.469**  **(*P*<0.001)** | - | **7.793**  **(*P*<0.001)** | - | **8.273**  **(*P*<0.001)** |
| FAQ | - | **1.122**  **(*P*<0.001)** | - | **1.113**  **(*P*<0.001)** | - | **1.090**  **(*P*<0.001)** | - | **1.103**  **(*P*<0.001)** | - | **1.100**  **(*P*<0.001)** | - | **1.100**  **(*P*<0.001)** |
| AIC/n | 0.955 | 0.874 | 0.776 | 0.672 | 0.772 | 0.650 | 0.750 | 0.636 | 0.830 | 0.686 | 0.930 | 0.747 |
| BIC/n | 0.966 | 0.905 | 0.792 | 0.708 | 0.788 | 0.692 | 0.765 | 0.672 | 0.845 | 0.733 | 0.946 | 0.789 |
| **Test accuracy** | | | | | | | | | | | | |
| AUC | 0.787 | 0.844 | 0.875 | 0.915 | 0.882 | 0.922 | 0.898 | 0.928 | 0.854 | 0.910 | 0.808 | 0.889 |
| AUC 95% CI | 0.754-0.820 | 0.817-0.871 | 0.851-0.900 | 0.896-0.934 | 0.858-0.907 | 0.903-0.941 | 0.878-0.922 | 0.911-0.946 | 0.827-0.881 | 0.890-0.931 | 0.777-0.839 | 0.866-0.912 |
| Threshold probability | 26.5^c^ | 0.221 | 0.305 | 0.251 | 0.304 | 0.293 | 0.326 | 0.375 | 0.253 | 0.231 | 0.251 | 0.361 |
| Sensitivity | 0.722 | 0.812 | 0.788 | 0.859 | 0.765 | 0.843 | 0.761 | 0.804 | 0.784 | 0.871 | 0.753 | 0.753 |
| Specificity | 0.727 | 0.731 | 0.819 | 0.813 | 0.853 | 0.877 | 0.899 | 0.902 | 0.783 | 0.802 | 0.740 | 0.875 |
| ^a^ Multiplied by 100 for ease of interpreting logistic regression coefficients.  ^b^ Excluded because the covariate was not significantly associated with outcome at 95% significance level.  ^c^ Value of MMSE rather than probability estimated by logistic regression.  **Abbreviation:** AIC: Akaike information criterion; AP: amyloid positivity; APOE4: apolipoprotein E allele 4; AUC: area under the curve; BIC: Bayesian information criterion; CI: confidence interval; FAQ: Functional Activities Questionnaire; GFAP: glial fibrillary acidic protein; MCI: mild cognitive impairment; MMSE: Mini-Mental State Examination; NfL: neurofilament light; PAD: probable Alzheimer’s disease. | | | | | | | | | | | | |


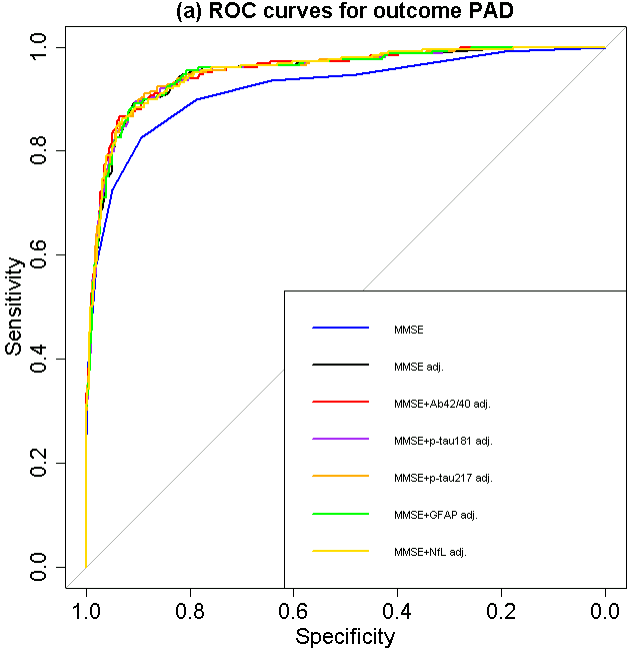

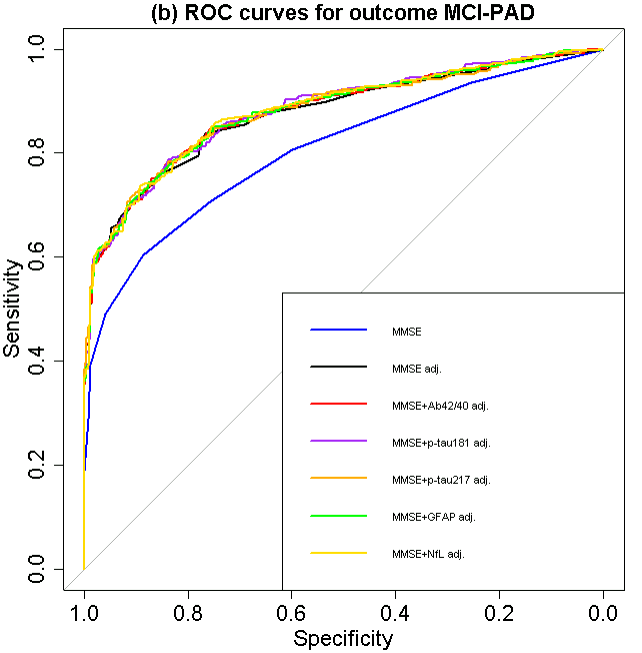

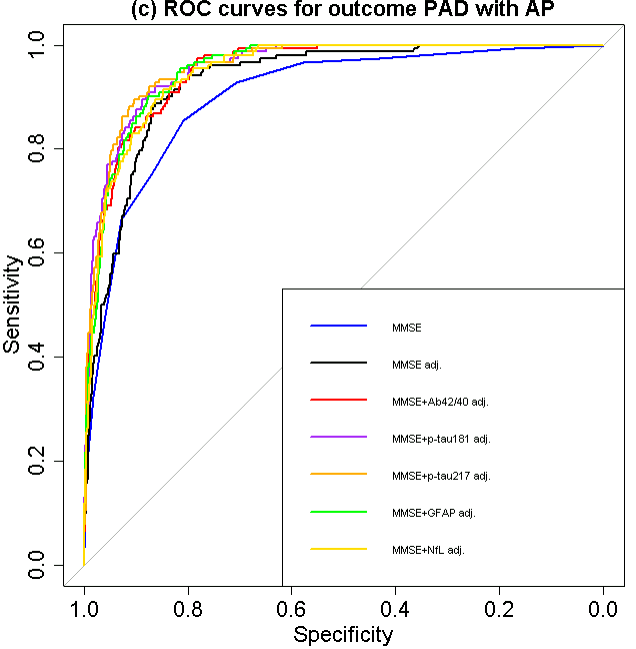

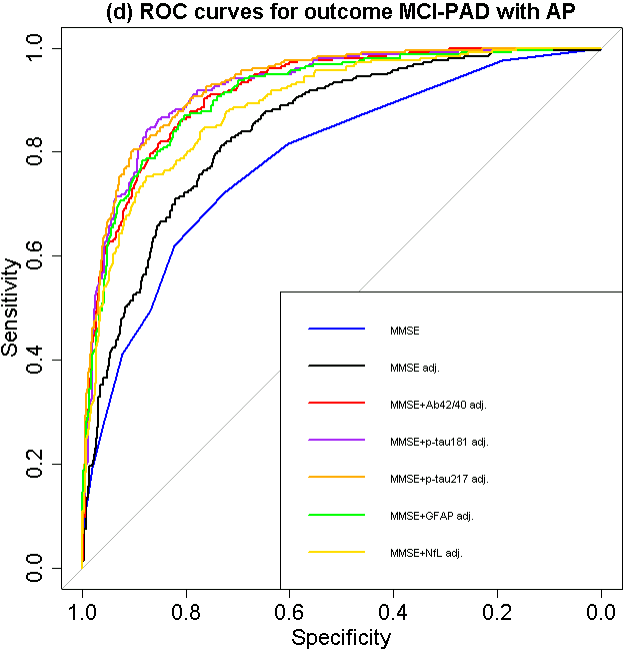


**eFigure 1** ROC curves for MMSE plus individual biomarkers by outcome. **Abbreviation:** adj.: adjusted; AP: amyloid positive; GFAP: glial fibrillary acidic protein; MCI: mild cognitive impairment; MMSE: Mini-Mental State Examination; NfL: neurofilament light; PAD: probable Alzheimer’s disease; ROC: receiver operating characteristic.

# Diagnostic accuracy of MMSE plus biomarker panels

| **eTable 7** Accuracy of MMSE plus blood biomarker panel. | | | | | | | | |
| --- | --- | --- | --- | --- | --- | --- | --- | --- |
|  | **Outcome** | | | | | | | |
|  | **PAD** | | **MCI-PAD** | | **PAD-AP** | | **MCI-PAD-AP** | |
|  | **MMSE + biomarker panel** | **MMSE + biomarker panel, adjusted** | **MMSE + biomarker panel** | **MMSE + biomarker panel, adjusted** | **MMSE + biomarker panel** | **MMSE + biomarker panel, adjusted** | **MMSE + biomarker panel** | **MMSE + biomarker panel, adjusted** |
| **Logistic regression odds ratios (bold if *P*<0.05). Sample n = 928** | | | | | | | | |
| Intercept | **2.973 e+7**  **(*P*<0.001)** | **8.265 e+3**  **(*P*<0.001)** | **9.096 e+5**  **(*P*<0.001)** | **5.996 e+4**  **(*P*<0.001)** | **6.141 e+7**  **(*P*<0.001)** | **2.161 e+2**  **(*P*=0.019)** | **2.723 e+6**  **(*P*<0.001)** | **1.811 e+3**  **(*P*=0.006)** |
| MMSE | **0.433**  **(*P*<0.001)** | **0.460**  **(*P*<0.001)** | **0.588**  **(*P*<0.001)** | **0.645**  **(*P*<0.001)** | **0.532**  **(*P*<0.001)** | **0.544**  **(*P*<0.001)** | **0.700**  **(*P*<0.001)** | **0.724**  **(*P*<0.001)** |
| Aβ42/40^a^ | **1.311**  **(*P*=0.029)** | **1.667**  **(*P*<0.001)** | Excluded | Excluded | **0.573**  **(*P*<0.001)** | Excluded | **0.389**  **(*P*<0.001)** | **0.454**  **(*P*<0.001)** |
| p-tau181 | Excluded^b^ | Excluded | Excluded | Excluded | **2.128**  **(*P*=0.017)** | **2.088**  **(*P*=0.037)** | **2.086**  **(*P=*0.006)** | **2.067**  **(*P=*0.018)** |
| p-tau217^a^ | **1.028**  **(*P*<0.001)** | **1.022**  **(*P*<0.001)** | **1.026**  **(*P*<0.001)** | **1.018**  **(*P*=0.011)** | **1.021**  **(*P*=0.007)** | **1.022**  **(*P*=0.009)** | **1.032**  **(*P*<0.001)** | **1.027**  **(*P*=0.002)** |
| GFAP | Excluded | Excluded | Excluded | Excluded | Excluded | Excluded | **1.007**  **(*P*=0.001)** | **1.007**  **(*P*=0.004)** |
| NfL | Excluded | Excluded | **1.172**  **(*P*<0.001)** | Excluded | Excluded | Excluded | Excluded | **0.867**  **(*P*=0.032)** |
| Age | - | **1.048**  **(*P*=0.021)** | - | Excluded | - | **1.068**  **(*P*=0.006)** | - | **1.053**  **(*P*=0.010)** |
| Female | - | Excluded | - | Excluded | - | Excluded | - | Excluded |
| Non-Hispanic White | - | Excluded | - | Excluded | - | Excluded | - | Excluded |
| Overweight/ obese | - | Excluded | - | Excluded | - | Excluded | - | Excluded |
| Alcohol use | - | Excluded | - | Excluded | - | Excluded | - | **1.953**  **(*P*=0.009)** |
| Tobacco use | - | Excluded | - | Excluded | - | **2.160**  **(*P*=0.009)** | - | Excluded |
| High blood pressure | - | Excluded | - | Excluded | - | Excluded | - | Excluded |
| Depression | - | Excluded | - | Excluded | - | Excluded | - | Excluded |
| APOE4 carrier | - | Excluded | - | Excluded | - | **5.838**  **(*P*<0.001)** | - | **5.022**  **(*P*<0.001)** |
| FAQ | - | **1.234**  **(*P*<0.001)** |  | **1.526**  **(*P*<0.001)** |  | **1.095**  **(*P*<0.001)** |  | **1.099**  **(*P*<0.001)** |
| AIC/n | 0.557 | 0.468 | 0.984 | 0.831 | 0.446 | 0.398 | 0.661 | 0.591 |
| BIC/n | 0.578 | 0.499 | 1.005 | 0.852 | 0.472 | 0.440 | 0.692 | 0.648 |
| **Test accuracy** | | | | | | | | |
| AUC | 0.927 | 0.954 | 0.825 | 0.879 | 0.941 | 0.959 | 0.919 | 0.939 |
| AUC 95% CI | 0.905-0.949 | 0.939-0.970 | 0.799-0.851 | 0.857-0.901 | 0.922-0.960 | 0.946-0.972 | 0.901-0.938 | 0.924-0.955 |
| Threshold probability | 0.248 | 0.324 | 0.661 | 0.625 | 0.151 | 0.161 | 0.317 | 0.219 |
| Sensitivity | 0.879 | 0.891 | 0.655 | 0.713 | 0.901 | 0.914 | 0.824 | 0.910 |
| Specificity | 0.869 | 0.919 | 0.893 | 0.911 | 0.854 | 0.885 | 0.878 | 0.845 |
| ^a^ Multiplied by 100 for ease of interpreting logistic regression coefficients.  ^b^ Excluded because the variable was not significantly associated with outcome at 95% significance level.  **Abbreviation:** AIC: Akaike information criterion; APOE4: apolipoprotein E allele 4; AUC: area under the curve; BIC: Bayesian information criterion; CI: confidence interval; FAQ: Functional Activities Questionnaire; GFAP: glial fibrillary acidic protein; MCI: mild cognitive impairment; MMSE: Mini-Mental State Examination; NfL: neurofilament light; PAD: probable Alzheimer’s disease. | | | | | | | | |

# Subgroup analyses

| **eTable 8** Accuracy of MMSE and blood biomarker panel to detect PAD. | | | | | | | | |
| --- | --- | --- | --- | --- | --- | --- | --- | --- |
|  | **Outcome: PAD** | | | | | | | |
|  | **Age ≤72** | | **Age >72** | | **Non-Hispanic White** | | **Other race/ethnicity** | |
|  | **MMSE, adjusted** | **MMSE + panel, adjusted** | **MMSE, adjusted** | **MMSE + panel, adjusted** | **MMSE, adjusted** | **MMSE + panel, adjusted** | **MMSE, adjusted** | **MMSE + panel, adjusted** |
| **Logistic regression odds ratios (bold if *P*<0.05)** | | | | | | | | |
| Intercept | **4.213 e+7**  **(*P*<0.001)** | **4.849 e+4**  **(*P*<0.001)** | **1.691 e+8**  **(*P*<0.001)** | **7.928 e+6**  **(*P*<0.001)** | **2.155 e+7**  **(*P*<0.001)** | **1.615 e+5**  **(*P*<0.001)** | **1.437 e+11**  **(*P*<0.001)** | **2.737 e+7**  **(*P*<0.001)** |
| MMSE | **0.460**  **(*P*<0.001)** | **0.469**  **(*P*<0.001)** | **0.449**  **(*P*<0.001)** | **0.443**  **(*P*<0.001)** | **0.478**  **(*P*<0.001)** | **0.485**  **(*P*<0.001)** | **0.351**  **(*P*<0.001)** | **0.346**  **(*P*<0.001)** |
| Aβ42/40^a^ | - | **1.707**  **(*P*=0.007)** | - | **1.419**  **(*P*=0.048)** | - | **1.511**  **(*P*=0.014)** | - | **2.056**  **(*P*=0.011)** |
| p-tau181 | - | Excluded | - | Excluded | - | Excluded | - | **5.195**  **(*P=*0.022)** |
| p-tau217^a^ | - | **1.038**  **(*P*<0.001)** | - | Excluded | - | **1.019**  **(*P*=0.004)** | - | Excluded |
| GFAP | - | Excluded | - | Excluded | - | Excluded | - | Excluded |
| NfL | - | Excluded | - | Excluded | - | Excluded | - | Excluded |
| Age | Excluded^b^ | Excluded | Excluded | Excluded | Excluded | Excluded | Excluded | Excluded |
| Female | Excluded | Excluded | Excluded | Excluded | Excluded | Excluded | Excluded | Excluded |
| Non-Hispanic White | Excluded | Excluded | Excluded | Excluded | - | - | - | - |
| Overweight/ obese | Excluded | Excluded | Excluded | Excluded | Excluded | Excluded | Excluded | Excluded |
| Alcohol use | Excluded | Excluded | Excluded | Excluded | Excluded | Excluded | **0.247**  **(*P*=0.007)** | **0.298**  **(*P*=0.024)** |
| Tobacco use | Excluded | Excluded | Excluded | Excluded | Excluded | Excluded | Excluded | Excluded |
| High blood pressure | Excluded | Excluded | Excluded | Excluded | Excluded | Excluded | Excluded | Excluded |
| Depression | Excluded | Excluded | Excluded | Excluded | Excluded | Excluded | Excluded | Excluded |
| APOE4 carrier | - | Excluded | - | Excluded | - | Excluded | - | Excluded |
| FAQ | **1.278**  **(*P*<0.001)** | **1.283**  **(*P*<0.001)** | **1.203**  **(*P*<0.001)** | **1.215**  **(*P*<0.001)** | **1.278**  **(*P*<0.001)** | **1.280**  **(*P*<0.001)** | **1.138**  **(*P*=0.007)** | **1.116**  **(*P*=0.031)** |
| Sample n | 478 | 478 | 450 | 450 | 710 | 710 | 218 | 218 |
| AIC/n | 0.400 | 0.373 | 0.582 | 0.577 | 0.458 | 0.447 | 0.546 | 0.524 |
| BIC/n | 0.427 | 0.416 | 0.609 | 0.614 | 0.477 | 0.479 | 0.608 | 0.617 |
| **Test accuracy** | | | | | | | | |
| AUC | 0.952 | 0.958 | 0.944 | 0.945 | 0.952 | 0.954 | 0.954 | 0.961 |
| AUC 95% CI | 0.926-0.977 | 0.934-0.983 | 0.922-0.967 | 0.923-0.968 | 0.933-0.971 | 0.935-0.973 | 0.926-0.983 | 0.936-0.986 |
| Threshold probability | 0.104 | 0.289 | 0.412 | 0.376 | 0.138 | 0.231 | 0.273 | 0.376 |
| Sensitivity | 0.943 | 0.885 | 0.876 | 0.876 | 0.953 | 0.917 | 0.949 | 0.924 |
| Specificity | 0.854 | 0.946 | 0.920 | 0.920 | 0.845 | 0.896 | 0.863 | 0.906 |
| ^a^ Multiplied by 100 for ease of interpreting logistic regression coefficients.  ^b^ Excluded because the variable was not significantly associated with outcome at 95% significance level.  **Abbreviation:** AIC: Akaike information criterion; APOE4: apolipoprotein E allele 4; AUC: area under the curve; BIC: Bayesian information criterion; CI: confidence interval; FAQ: Functional Activities Questionnaire; GFAP: glial fibrillary acidic protein; MMSE: Mini-Mental State Examination; NfL: neurofilament light; PAD: probable Alzheimer’s disease. | | | | | | | | |

| **eTable 9** Accuracy of MMSE and blood biomarker panel to detect MCI-PAD. | | | | | | | | |
| --- | --- | --- | --- | --- | --- | --- | --- | --- |
|  | **Outcome: MCI-PAD** | | | | | | | |
|  | **Age ≤72** | | **Age >72** | | **Non-Hispanic White** | | **Other race/ethnicity** | |
|  | **MMSE, adjusted** | **MMSE + panel, adjusted** | **MMSE, adjusted** | **MMSE + panel, adjusted** | **MMSE, adjusted** | **MMSE + panel, adjusted** | **MMSE, adjusted** | **MMSE + panel, adjusted** |
| **Logistic regression odds ratios (bold if *P*<0.05)** | | | | | | | | |
| Intercept | **6.602 e+4**  **(*P*<0.001)** | **6.602 e+4**  **(*P*<0.001)** | **2.316 e+5**  **(*P*<0.001)** | **1.299 e+5**  **(*P*<0.001)** | **3.466 e+4**  **(*P*<0.001)** | **1.480 e+4**  **(*P*<0.001)** | **1.990 e+6**  **(*P*<0.001)** | **7.381 e+5**  **(*P*<0.001)** |
| MMSE | **0.647**  **(*P*<0.001)** | **0.647**  **(*P*<0.001)** | **0.615**  **(*P*<0.001)** | **0.619**  **(*P*<0.001)** | **0.665**  **(*P*<0.001)** | **0.678**  **(*P*<0.001)** | **0.573**  **(*P*<0.001)** | **0.575**  **(*P*<0.001)** |
| Aβ42/40^a^ | - | Excluded | - | Excluded | - | Excluded | - | Excluded |
| p-tau181 | - | Excluded | - | Excluded | - | Excluded | - | Excluded |
| p-tau217^a^ | - | Excluded | - | **1.023**  **(*P*=0.014)** | - | **1.019**  **(*P*=0.013)** | - | Excluded |
| GFAP | - | Excluded | - | Excluded | - | Excluded | - | Excluded |
| NfL | - | Excluded | - | Excluded | - | Excluded | - | **1.459**  **(*P*=0.027)** |
| Age | Excluded^b^ | Excluded | Excluded | Excluded | Excluded | Excluded | Excluded | Excluded |
| Female | Excluded | Excluded | Excluded | Excluded | Excluded | Excluded | Excluded | Excluded |
| Non-Hispanic White | Excluded | Excluded | Excluded | Excluded | - | - | - | - |
| Overweight/ obese | Excluded | Excluded | Excluded | Excluded | Excluded | Excluded | Excluded | Excluded |
| Alcohol use | Excluded | Excluded | **1.906**  **(*P*=0.032)** | **1.832**  **(*P*=0.046)** | Excluded | Excluded | Excluded | Excluded |
| Tobacco use | Excluded | Excluded | Excluded | Excluded | Excluded | Excluded | Excluded | Excluded |
| High blood pressure | Excluded | Excluded | Excluded | Excluded | Excluded | Excluded | Excluded | Excluded |
| Depression | Excluded | Excluded | Excluded | Excluded | Excluded | Excluded | Excluded | Excluded |
| APOE4 carrier | - | Excluded | - | Excluded | - | Excluded | - | Excluded |
| FAQ | **1.683**  **(*P*<0.001)** | **1.683**  **(*P*<0.001)** | **1.462**  **(*P*<0.001)** | **1.424**  **(*P*<0.001)** | **1.575**  **(*P*<0.001)** | **1.533**  **(*P*<0.001)** | **1.532**  **(*P*<0.001)** | **1.495**  **(*P*<0.001)** |
| Sample n | 478 | 478 | 450 | 450 | 710 | 710 | 218 | 218 |
| AIC/n | 0.878 | 0.878 | 0.794 | 0.783 | 0.867 | 0.860 | 0.760 | 0.745 |
| BIC/n | 0.904 | 0.904 | 0.831 | 0.829 | 0.886 | 0.885 | 0.806 | 0.807 |
| **Test accuracy** | | | | | | | | |
| AUC | 0.861 | 0.861 | 0.887 | 0.892 | 0.871 | 0.872 | 0.904 | 0.909 |
| AUC 95% CI | 0.827-0.895 | 0.827-0.895 | 0.857-0.917 | 0.863-0.921 | 0.845-0.897 | 0.846-0.898 | 0.866-0.943 | 0.871-0.946 |
| Threshold probability | 0.477 | 0.477 | 0.717 | 0.694 | 0.480 | 0.414 | 0.669 | 0.657 |
| Sensitivity | 0.742 | 0.742 | 0.714 | 0.741 | 0.774 | 0.826 | 0.778 | 0.806 |
| Specificity | 0.873 | 0.873 | 0.933 | 0.933 | 0.847 | 0.784 | 0.905 | 0.905 |
| ^a^ Multiplied by 100 for ease of interpreting logistic regression coefficients.  ^b^ Excluded because the variable was not significantly associated with outcome at 95% significance level.  **Abbreviation:** AIC: Akaike information criterion; APOE4: apolipoprotein E allele 4; AUC: area under the curve; BIC: Bayesian information criterion; CI: confidence interval; FAQ: Functional Activities Questionnaire; GFAP: glial fibrillary acidic protein; MCI: mild cognitive impairment; MMSE: Mini-Mental State Examination; NfL: neurofilament light; PAD: probable Alzheimer’s disease. | | | | | | | | |

| **eTable 10** Accuracy of MMSE and blood biomarker panel to detect PAD with AP. | | | | | | | | |
| --- | --- | --- | --- | --- | --- | --- | --- | --- |
|  | **Outcome: PAD-AP** | | | | | | | |
|  | **Age ≤72** | | **Age >72** | | **Non-Hispanic White** | | **Other race/ethnicity** | |
|  | **MMSE, adjusted** | **MMSE + panel, adjusted** | **MMSE, adjusted** | **MMSE + panel, adjusted** | **MMSE, adjusted** | **MMSE + panel, adjusted** | **MMSE, adjusted** | **MMSE + panel, adjusted** |
| **Logistic regression odds ratios (bold if *P*<0.05)** | | | | | | | | |
| Intercept | **1.702 e+5**  **(*P*<0.001)** | **1.062 e+5**  **(*P*<0.001)** | **2.755 e+5**  **(*P*<0.001)** | **2.978 e+4**  **(*P*<0.001)** | **3.673 e+3**  **(*P*<0.001)** | 1.674 e+2  (*P*=0.065) | **1.876 e+5**  **(*P*<0.001)** | **4.573 e+5**  **(*P*<0.001)** |
| MMSE | **0.554**  **(*P*<0.001)** | **0.489**  **(*P*<0.001)** | **0.549**  **(*P*<0.001)** | **0.580**  **(*P*<0.001)** | **0.519**  **(*P*<0.001)** | **0.520**  **(*P*<0.001)** | **0.552**  **(*P*<0.001)** | **0.470**  **(*P*<0.001)** |
| Aβ42/40^a^ | - | Excluded | - | Excluded | - | Excluded | - | Excluded |
| p-tau181 | - | Excluded | - | Excluded | - | Excluded | - | **1.727**  **(*P*<0.001)** |
| p-tau217^a^ | - | **1.036**  **(*P*<0.001)** | - | **1.034**  **(*P*<0.001)** | - | **1.032**  **(*P*<0.001)** | - | Excluded |
| GFAP | - | Excluded | - | Excluded | - | Excluded | - | Excluded |
| NfL | - | Excluded | - | Excluded | - | Excluded | - | Excluded |
| Age | Excluded^b^ | Excluded | Excluded | Excluded | **1.078**  **(*P*=0.002)** | **1.099**  **(*P*<0.001)** | Excluded | Excluded |
| Female | Excluded | Excluded | Excluded | Excluded | Excluded | Excluded | Excluded | **0.283**  **(*P*=0.028)** |
| Non-Hispanic White | Excluded | Excluded | **2.755**  **(*P*=0.009)** | Excluded | - | - | - | - |
| Overweight/ obese | Excluded | Excluded | Excluded | Excluded | Excluded | Excluded | Excluded | Excluded |
| Alcohol use | Excluded | Excluded | Excluded | Excluded | **2.346**  **(*P*=0.016)** | Excluded | Excluded | Excluded |
| Tobacco use | Excluded | **3.592**  **(*P*=0.021)** | Excluded | Excluded | **1.841**  **(*P*=0.047)** | **2.523**  **(*P*=0.008)** | Excluded | Excluded |
| High blood pressure | Excluded | Excluded | Excluded | Excluded | **0.433**  **(*P*=0.012)** | Excluded | Excluded | Excluded |
| Depression | Excluded | Excluded | Excluded | Excluded | Excluded | Excluded | Excluded | Excluded |
| APOE4 carrier | - | **16.635**  **(*P*<0.001)** | - | **3.728**  **(*P*<0.001)** | - | **6.314**  **(*P*<0.001)** | - | **5.698**  **(*P*=0.003)** |
| FAQ | **1.152**  **(*P*<0.001)** | **1.163**  **(*P*<0.001)** | **1.076**  **(*P*=0.004)** | **1.072**  **(*P*=0.014)** | **1.123**  **(*P*<0.001)** | **1.092**  **(*P*=0.002)** | **1.114**  **(*P*=0.004)** | Excluded |
| Sample n | 478 | 478 | 450 | 450 | 710 | 710 | 218 | 218 |
| AIC/n | 0.357 | 0.261 | 0.669 | 0.564 | 0.465 | 0.381 | 0.591 | 0.443 |
| BIC/n | 0.383 | 0.313 | 0.705 | 0.609 | 0.510 | 0.426 | 0.638 | 0.521 |
| **Test accuracy** | | | | | | | | |
| AUC | 0.943 | 0.974 | 0.905 | 0.937 | 0.937 | 0.961 | 0.914 | 0.956 |
| AUC 95% CI | 0.914-0.972 | 0.960-0.989 | 0.875-0.936 | 0.913-0.960 | 0.916-0.958 | 0.946-0.976 | 0.876-0.953 | 0.927-0.986 |
| Threshold probability | 0.110 | 0.122 | 0.190 | 0.285 | 0.159 | 0.104 | 0.119 | 0.256 |
| Sensitivity | 0.915 | 0.936 | 0.895 | 0.848 | 0.893 | 0.946 | 0.975 | 0.900 |
| Specificity | 0.879 | 0.923 | 0.806 | 0.890 | 0.856 | 0.848 | 0.764 | 0.921 |
| ^a^ Multiplied by 100 for ease of interpreting logistic regression coefficients.  ^b^ Excluded because the variable was not significantly associated with outcome at 95% significance level.  **Abbreviation:** AIC: Akaike information criterion; AP: amyloid positivity; APOE4: apolipoprotein E allele 4; AUC: area under the curve; BIC: Bayesian information criterion; CI: confidence interval; FAQ: Functional Activities Questionnaire; GFAP: glial fibrillary acidic protein; MMSE: Mini-Mental State Examination; NfL: neurofilament light; PAD: probable Alzheimer’s disease. | | | | | | | | |

| **eTable 11** Accuracy of MMSE and blood biomarker panel to detect MCI-PAD with AP. | | | | | | | | |
| --- | --- | --- | --- | --- | --- | --- | --- | --- |
|  | **Outcome: MCI-PAD-AP** | | | | | | | |
|  | **Age ≤72** | | **Age >72** | | **Non-Hispanic White** | | **Other race/ethnicity** | |
|  | **MMSE, adjusted** | **MMSE + panel, adjusted** | **MMSE, adjusted** | **MMSE + panel, adjusted** | **MMSE, adjusted** | **MMSE + panel, adjusted** | **MMSE, adjusted** | **MMSE + panel, adjusted** |
| **Logistic regression odds ratios (bold if *P*<0.05)** | | | | | | | | |
| Intercept | 0.264  (*P*=0.684) | **1.823 e+6**  **(*P*<0.001)** | **4.765 e+2**  **(*P*<0.001)** | **8.224 e+4**  **(*P*<0.001)** | **3.694 e+3**  **(*P*<0.001)** | **1.523 e+3**  **(*P*=0.024)** | **1.773 e+2**  **(*P*=0.002)** | **5.880 e+4**  **(*P*=0.005)** |
| MMSE | **0.735**  **(*P*<0.001)** | **0.695**  **(*P*<0.001)** | **0.723**  **(*P*<0.001)** | **0.731**  **(*P*<0.001)** | **0.684**  **(*P*<0.001)** | **0.675**  **(*P*<0.001)** | **0.760**  **(*P*<0.001)** | **0.748**  **(*P*<0.001)** |
| Aβ42/40^a^ | - | **0.375**  **(*P*<0.001)** | - | **0.446**  **(*P*<0.001)** | - | **0.449**  **(*P*<0.001)** | - | **0.426**  **(*P*=0.003)** |
| p-tau181 | - | Excluded | - | Excluded | - | Excluded | - | **7.181**  **(*P<*0.001)** |
| p-tau217^a^ | - | **1.042**  **(*P*<0.001)** | - | **1.043**  **(*P*<0.001)** | - | **1.043**  **(*P*<0.001)** | - | Excluded |
| GFAP | - | Excluded | - | **1.011**  **(*P*=0.002)** | - | **1.009**  **(*P*=0.004)** | - | Excluded |
| NfL | - | Excluded | - | **0.827**  **(*P*=0.031)** | - | **0.794**  **(*P*=0.011)** | - | Excluded |
| Age | **1.117**  **(*P*=0.008)** | Excluded | Excluded | Excluded | Excluded | **1.089**  **(*P*<0.001)** | Excluded | Excluded |
| Female | Excluded | Excluded | Excluded | Excluded | Excluded | Excluded | Excluded | Excluded |
| Non-Hispanic White | Excluded | Excluded | **2.171**  **(*P*=0.015)** | Excluded | - | - | - | - |
| Overweight/ obese | Excluded | Excluded | Excluded | Excluded | Excluded | Excluded | Excluded | Excluded |
| Alcohol use | Excluded | Excluded | **2.398**  **(*P*<0.001)** | **3.421**  **(*P*<0.001)** | **2.256**  **(*P*=0.001)** | **2.835**  **(*P*=0.001)** | Excluded | Excluded |
| Tobacco use | Excluded | Excluded | Excluded | Excluded | Excluded | Excluded | Excluded | Excluded |
| High blood pressure | Excluded | Excluded | Excluded | Excluded | **0.532**  **(*P*=0.006)** | Excluded | Excluded | Excluded |
| Depression | Excluded | Excluded | Excluded | **2.137**  **(*P*=0.020)** | Excluded | Excluded | Excluded | Excluded |
| APOE4 carrier | - | **10.770**  **(*P*<0.001)** | - | **3.552**  **(*P*<0.001)** | - | **5.714**  **(*P*<0.001)** | - | **4.882**  **(*P*=0.001)** |
| FAQ | **1.153**  **(*P*<0.001)** | **1.139**  **(*P*<0.001)** | **1.089**  **(*P*<0.001)** | **1.078**  **(*P*=0.011)** | **1.117**  **(*P*<0.001)** | **1.083**  **(*P*=0.003)** | **1.140**  **(*P*<0.001)** | **1.137**  **(*P*=0.005)** |
| Sample n | 478 | 478 | 450 | 450 | 710 | 710 | 218 | 218 |
| AIC/n | 0.697 | 0.457 | 1.061 | 0.740 | 0.877 | 0.576 | 0.917 | 0.636 |
| BIC/n | 0.732 | 0.509 | 1.107 | 0.831 | 0.909 | 0.640 | 0.964 | 0.729 |
| **Test accuracy** | | | | | | | | |
| AUC | 0.865 | 0.953 | 0.805 | 0.923 | 0.840 | 0.943 | 0.824 | 0.932 |
| AUC 95% CI | 0.823-0.906 | 0.932-0.973 | 0.763-0.847 | 0.898-0.947 | 0.808-0.873 | 0.926-0.960 | 0.763-0.886 | 0.898-0.967 |
| Threshold probability | 0.197 | 0.162 | 0.319 | 0.222 | 0.242 | 0.223 | 0.270 | 0.309 |
| Sensitivity | 0.773 | 0.943 | 0.749 | 0.922 | 0.765 | 0.903 | 0.746 | 0.864 |
| Specificity | 0.841 | 0.864 | 0.728 | 0.784 | 0.772 | 0.844 | 0.805 | 0.874 |
| ^a^ Multiplied by 100 for ease of interpreting logistic regression coefficients.  ^b^ Excluded because the variable was not significantly associated with outcome at 95% significance level.  **Abbreviation:** AIC: Akaike information criterion; AP: amyloid positivity; APOE4: apolipoprotein E allele 4; AUC: area under the curve; BIC: Bayesian information criterion; CI: confidence interval; FAQ: Functional Activities Questionnaire; GFAP: glial fibrillary acidic protein; MCI: mild cognitive impairment; MMSE: Mini-Mental State Examination; NfL: neurofilament light; PAD: probable Alzheimer’s disease. | | | | | | | | |

# References

1. Mohs RC, Beauregard D, Dwyer J, Gaudioso J, Bork J, MaGee‐Rodgers T, et al. The Bio‐Hermes Study: Biomarker database developed to investigate blood‐based and digital biomarkers in community‐based, diverse populations clinically screened for Alzheimer's disease. Alzheimer's & Dementia. 2024;20(4):2752-65.

2. Zimek A, Filzmoser P. There and back again: Outlier detection between statistical reasoning and data mining algorithms. Wiley Interdisciplinary Reviews: Data Mining and Knowledge Discovery. 2018;8(6):e1280.

3. National Institute for Health and Care Excellence. Hypertension: What is it? 2023. Available from: <https://cks.nice.org.uk/topics/hypertension/background-information/definition/>.

4. Greenberg SA. The Geriatric Depression Scale (GDS) 2023. Available from: <https://hign.org/consultgeri/try-this-series/geriatric-depression-scale-gds>.
